# Octopaminergic Signaling Mediates Neural Regulation of Innate Immunity in Caenorhabditis elegans

**DOI:** 10.1128/mBio.01645-18

**Published:** 2018-10-09

**Authors:** Durai Sellegounder, Chung-Hsiang Yuan, Phillip Wibisono, Yiyong Liu, Jingru Sun

**Affiliations:** a Department of Biomedical Sciences, Elson S. Floyd College of Medicine, Washington State University, Spokane, Washington, USA; b Genomics Core, Washington State University, Spokane, Washington, USA; Mass General Hospital

**Keywords:** *Caenorhabditis elegans*, G protein-coupled receptor, innate immunity, neural regulation, octopamine

## Abstract

Upon pathogen infection, the nervous system regulates innate immunity to confer coordinated protection to the host. However, the precise mechanisms of such regulation remain unclear. Previous studies have demonstrated that OCTR-1, a putative G protein-coupled receptor for catecholamine, functions in the sensory neurons designated “ASH” to suppress innate immune responses in Caenorhabditis elegans. It is unknown what molecules act as OCTR-1 ligands in the neural immune regulatory circuit. Here we identify neurotransmitter octopamine (OA) as an endogenous ligand for OCTR-1 in immune regulation and show that the OA-producing RIC neurons function in the OCTR-1 neural circuit to suppress innate immunity. RIC neurons are deactivated in the presence of pathogens but transiently activated by nonpathogenic bacteria. Our data support a model whereby an octopaminergic immunoinhibitory pathway is tonically active under normal conditions to maintain immunological homeostasis or suppress unwanted innate immune responses but downregulated upon pathogen infection to allow enhanced innate immunity. As excessive innate immune responses have been linked to a myriad of human health concerns, our study could potentially benefit the development of more-effective treatments for innate immune disorders.

## Abstract (precis)

Insufficient or excessive immune responses to pathogen infection are major causes of disease. Increasing evidence indicates that the nervous system regulates the immune system to help maintain immunological homeostasis. However, the precise mechanisms of this regulation are largely unknown. Here we show the existence of an octopaminergic immunoinhibitory pathway in Caenorhabditis elegans. Our study results indicate that this pathway is tonically active under normal conditions to maintain immunological homeostasis or suppress unwanted innate immune responses but downregulated upon pathogen infection to allow enhanced innate immunity. As excessive innate immune responses have been linked to human health conditions such as Crohn's disease, rheumatoid arthritis, atherosclerosis, diabetes, and Alzheimer's disease, elucidating octopaminergic neural regulation of innate immunity could be helpful in the development of new treatments for innate immune diseases.

## INTRODUCTION

Upon pathogen infection, cellular stress pathways and microbial killing pathways are rapidly activated by the host innate immune system. These pathways must be tightly regulated as insufficient immune responses exacerbate infection, whereas excessive immune responses lead to prolonged inflammation, tissue damage, or even death (1). Mammalian studies indicate that the nervous system regulates the immune system to help maintain immunological homeostasis (1–3). However, the functions of individual neurons and neural circuits in this process remain challenging to study and largely unexplored due to the complexity of the mammalian nervous system (an adult human brain contains about 86 billion neurons [4]). With current technology, it is difficult to dissect neural immune signaling at the neuronal level in mammals. The nematode Caenorhabditis elegans is an excellent model organism for such studies due to its possessing a simple, well-defined nervous system and an immune system that resembles the human innate immune system in several key respects (5, 6). C. elegans has only 302 neurons; the identity, morphology, and synaptic connectivity of each neuron are well described. It is the only animal for which the synaptic wiring diagram of the nervous system has been completely established (7). Also, most gene families involved in mammalian neuronal functions are found in C. elegans (8). Moreover, upon infection with microorganisms, including many human pathogens, C. elegans can mount innate immune responses by activating signaling pathways that are conserved in humans (9–11). Application of the C. elegans model system to study neural immune signaling has greatly facilitated our understanding of neural immune regulatory circuits (12–20).

Recent studies in C. elegans have led to the identification of specific G protein-coupled receptors (GPCRs), neurotransmitters, neuropeptides, neurons, and nonneural cells in the regulation of innate immunity (12–20). Worms deficient in NPR-1, a homologue of the neuropeptide Y receptor in mammals, show a decrease in pathogen avoidance and decreased innate immune responses to a number of bacterial pathogens (15, 21). Anderson et al. (16) demonstrated that, upon infection of C. elegans with the bacterial pathogen *Microbacterium nematophilum*, the neurotransmitter serotonin acts via its receptors SER-1 and SER-7 to suppress innate immune responses in the rectal epithelium. Chemical inhibition of the neurotransmitter dopamine in C. elegans CEP neurons enhances a microbicidal PMK-1/p38 mitogen-activated protein kinase (MAPK) signaling pathway (17). Pathogen infection induces secretion of neuropeptides INS-7 and DBL-1 from the nervous system that regulates the expression of immune genes in nonneural tissues (19, 20, 22). We demonstrated that OCTR-1, a putative GPCR for catecholamine expressed in the cilia of neurons located in sensory openings (23), functions in ASH and ASI sensory neurons to suppress innate immune responses in intestine and pharynx in a cell-non-autonomous manner (12–14). Aballay and colleagues (24) further pinpointed the finding that ASH neurons control innate immunity whereas ASI neurons promote pathogen avoidance behavior. The OCTR-1-dependent immune regulation is achieved by downregulating gene expression of the PMK-1/p38 MAPK immune pathway and the unfolded protein response (UPR) pathways (12–14). It is not clear what ligand(s) activates OCTR-1 in immune regulation or how the OCTR-1 pathway operates.

OCTR-1 was initially identified as a receptor for the neurotransmitter octopamine (OA) in behavioral responses of C. elegans to chemical stimulation (23). It is unknown what role the OA-OCTR-1 interaction plays in innate immune modulation against pathogen infection. OA is primarily an invertebrate nonpeptide transmitter that is structurally and physiologically related to the vertebrate neurotransmitter norepinephrine (25, 26). In insects, OA is involved in cellular immune responses (27–29). Here we demonstrate that both endogenous OA and exogenous OA inhibit immune responses in C. elegans and identify OA as an endogenous ligand for OCTR-1 in immune regulation. The OA-producing RIC neurons function in the OCTR-1 neural circuit to suppress innate immunity. RICs are deactivated in the presence of the human pathogen Pseudomonas aeruginosa strain PA14, whereas they are transiently activated in the presence of the nonpathogenic bacterium Escherichia coli strain OP50. A model emerges whereby an octopaminergic immunoinhibitory pathway is tonically active under normal conditions to maintain immunological homeostasis or suppress unwanted innate immune responses but downregulated upon pathogen infection to allow enhanced innate immunity.

## RESULTS

### OA regulates innate immunity in C. elegans.

**FIG 1:**
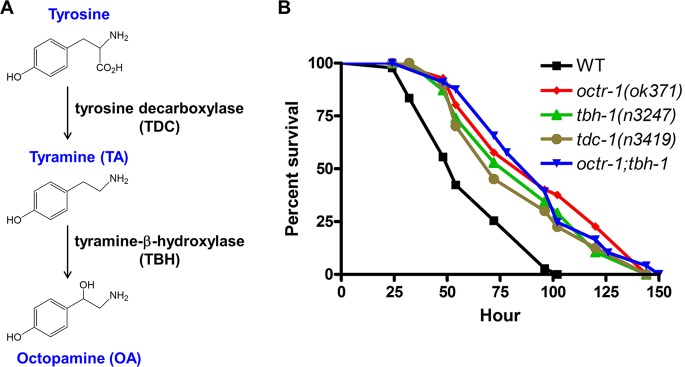
OA is involved in the resistance of C. elegans against P. aeruginosa infection. **(**A) Biosynthetic pathway of OA. OA is produced from tyramine (TA) through tyramine-β-hydroxylase (TBH); TA is the direct decarboxylation product of tyrosine through tyrosine decarboxylase (TDC). (B) Wild-type (WT) N2, *octr-1*(*ok371*), *tbh-1*(*n3247*), *tdc-1*(*n3419*), and *octr-1*(*ok371*)*;tbh-1*(*n3247*) animals were exposed to P. aeruginosa and scored for survival over time. *P* values represent results of comparisons to WT animals as follows: *octr-1*(*ok371*) (*P < *0.0001); *tbh-1*(*n3247*) (*P < *0.0001); *tdc-1*(*n3419*) (*P < *0.0001); *octr-1*(*ok371*)*;tbh-1*(*n3247*) (*P < *0.0001). The survival graph is a representative of results from three independent experiments. *n* = 45 to 60 animals per strain per experiment.

OA is a monoamine that is produced from tyramine (TA) through tyramine-β-hydroxylase (TBH); and TA is the direct decarboxylation product of tyrosine through tyrosine decarboxylase (TDC) (Fig. 1A). Invertebrate OA and TA are structurally related to noradrenaline and adrenaline in vertebrates and have similar physiological roles, indicating an early evolutionary origin of the adrenergic/octopaminergic/tyraminergic system (25, 26). OA and TA act independently through GPCRs to modulate multiple physiological and behavioral processes in response to external stimuli (25). Without the TDC enzyme, neither TA nor OA is produced. If TBH is knocked out, OA is eliminated and TA accumulates (23, 30). To determine if OA is involved in innate immunity against pathogen infection, we exposed the following strains of adult animals to human-pathogenic Pseudomonas aeruginosa strain PA14 and scored the nematode’s survival over time: wild-type N2, C. elegans lacking OCTR-1 [*octr-1*(*ok371*) null animals], TBH [*tbh-1*(*n3247*) null animals], TDC [*tdc-1*(*n3419*) null animals], or both OCTR-1 and TBH [*octr-1*(*ok371*)*;tbh-1*(*n3247*)]. Consistent with an inhibitory role of OCTR-1 in immunity (12, 13), *octr-1*(*ok371*) animals exhibited an enhanced resistance phenotype (*erp*) to P. aeruginosa-mediated killing compared to wild-type animals (Fig. 1B). Similarly to *octr-1*(*ok371*) animals, both *tbh-1*(*n3247*) and *tdc-1*(*n3419*) animals showed *erp* with respect to P. aeruginosa (Fig. 1B). When feeding on the standard food source Escherichia coli OP50, these mutant animals have a life span comparable to that of wild-type animals (31). These results indicate that lack of TBH or TDC protects C. elegans from pathogen infection without affecting its life span.

**FIG 2:**
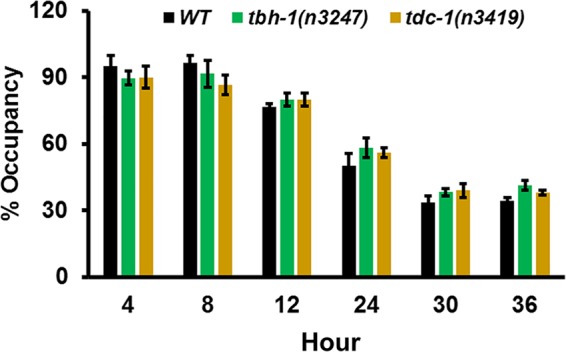
Bacterial lawn avoidance assays showed similar P. aeruginosa avoidance by wild-type and mutant animals. Wild-type N2 and *tbh-1*(*n3247*) and *tdc-1*(*n3419*) animals were placed on a small spot of P. aeruginosa on a 3.5-cm-diameter plate and monitored over time for their presence on the lawn. Bars represent means ± standard errors of the means (SEM). *n* = 3 independent experiments.

The enhanced survival of the mutant animals against P. aeruginosa challenge could be due to increased pathogen avoidance, because avoidance behavior is part of the C. elegans defense response to P. aeruginosa (15). To test this possibility, we measured C. elegans survival using full-lawn assays in which agar plates were completely covered in bacteria such that pathogen avoidance was eliminated. Both *tbh-1*(*n3247*) and *tdc-1*(*n3419*) animals died at a lower rate than the wild-type animals did (see Fig. S1 in the supplemental material). Additionally, bacterial lawn avoidance assays, in which small lawns of P. aeruginosa were cultured in the center of agar plates and the numbers of animals that stayed on and off the lawn were counted at six time points over a period of 36 h, showed that the magnitudes of pathogen avoidance of the mutants and wild-type animals were similar (Fig. 2). These results indicate that pathogen avoidance does not play a role in the enhanced survival of the mutant animals.

10.1128/mBio.01645-18.2 FIG S1 Full-lawn assays to measure survival of *tbh-1*(*n3247*), *tdc-1*(*n3419*), and wild-type animals against P. aeruginosa. Download FIG S1, TIF file, 0.2 MB. Copyright © 2018 Sellegounder et al. 2018 Sellegounder et al. This content is distributed under the terms of the Creative Commons Attribution 4.0 International license.

**FIG 3:**
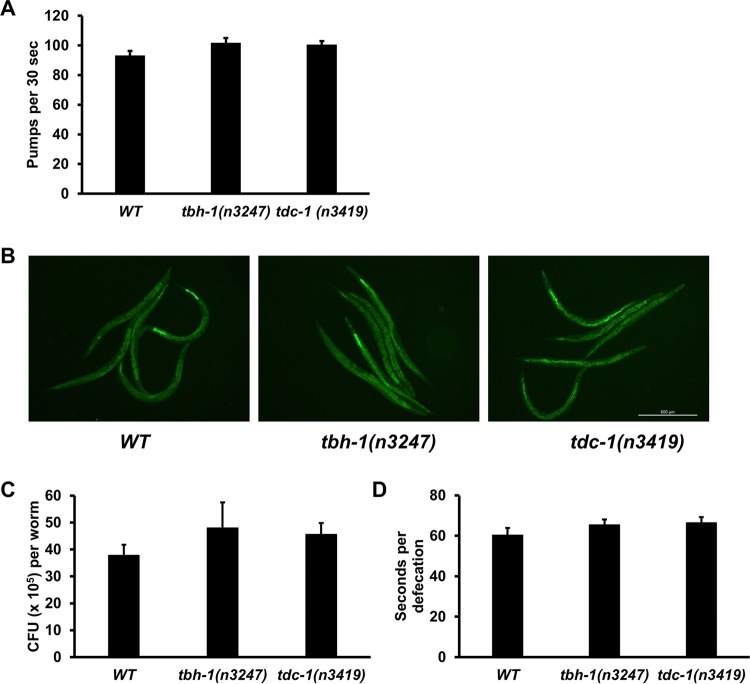
The levels of P. aeruginosa intake or accumulation are similar among wild-type, *tbh-1*(*n3247*), and *tdc-1*(*n3419*) animals. (A) Pharyngeal pumping rates of animals on P. aeruginosa lawns were counted as pumps per 30 s. Counting was conducted in triplicate, and results were averaged to give a pumping rate. The graph represents combined results of three independent experiments. Bars represent means ± SEM. *n* = 30 animals per strain. *P* values represent results of comparisons to WT animals as follows: *tbh-1*(*n3247*) (*P = *0.1270); *tdc-1*(*n3419*) (*P = *0.1231). (B) Animals were exposed to P. aeruginosa expressing GFP for 24 h and then visualized using a Leica MZ10F fluorescence stereomicroscope. Scale bar indicates 500 μm. (C) Animals were exposed to P. aeruginosa expressing GFP for 24 h at 25°C, and CFU counts were performed. Ten animals were used for each condition. The graph represents combined results of three independent experiments. Bars represent means ± SEM. *P* values represent results of comparisons to WT animals as follows: *tbh-1*(*n3247*) (*P = *0.3803); *tdc-1*(*n3419*) (*P = *0.2426). (D) Defecation rates of animals on P. aeruginosa lawns were measured as averages of 10 intervals between two defecation cycles. Bars represent means ± SEM. *n* = 12 animals per strain. *P* values represent results of comparisons to WT animals as follows: *tbh-1*(*n3247*) (*P = *0.2575); *tdc-1*(*n3419*) (*P = *0.1838).

The altered survival of *tbh-1*(*n3247*) and *tdc-1*(*n3419*) animals against P. aeruginosa could have been due to a change in immunity and/or a change in pathogen intake or accumulation. C. elegans feeds on bacterial food via rhythmic contractions (pumping) of its pharynx. To determine if there is any difference in pathogen intake between these mutants and wild-type animals, we measured the pharyngeal pumping rates of the animals on bacterium lawns. When feeding on P. aeruginosa, the two types of mutant animals showed pumping rates similar to those seen with wild-type animals (Fig. 3A), indicating similar levels of pathogen intake. Because certain mutations in C. elegans can cause reduced pathogen accumulation in the intestine, resulting in enhanced resistance to pathogen infection (32), we then examined whether *tbh-1* or *tdc-1* mutation affects bacterial accumulation. The mutants and wild-type animals showed similar accumulation patterns of P. aeruginosa/green fluorescent protein (GFP) (Fig. 3B). Intestinal bacterial loads were quantified by determining CFU counts of live bacterial cells recovered from the intestine (12). Both types of mutant animals showed slightly but insignificantly more CFU of P. aeruginosa than wild-type animals (Fig. 3C), indicating that *tbh-1* or *tdc-1* mutation did not cause a reduction in bacterial accumulation. We also examined if the mutations affected bacterial evacuation from the animals. The rate of bacterial evacuation can be estimated by measuring the defecation rate, defined as the time interval between expulsions of gut contents. The defecation cycle interval of the two types of mutant animals on P. aeruginosa was similar to that seen with wild-type animals (Fig. 3D). These results suggest that the improved survival of *tbh-1*(*n3247*) and *tdc-1*(*n3419*) animals against P. aeruginosa was not due to a change in pathogen intake or accumulation. Therefore, we attribute their improved survival to enhanced innate immunity. Because *tbh-1*(*n3247*) animals lack OA but still make TA, these results indicate that OA is involved in the regulation of immunity against P. aeruginosa, whereas TA is not. No additive survival advantage was observed in the double mutant *octr-1*(*ok371*)*;tbh-1*(*n3247*) compared to single mutant *octr-1*(*ok371*) or single mutant *tbh-1*(*n3247*) (Fig. 1B), suggesting that TBH and OCTR-1 could function in the same signaling pathway that suppresses innate immunity.

### OA functions in OCTR-1-dependent innate immunity.

**FIG 4:**
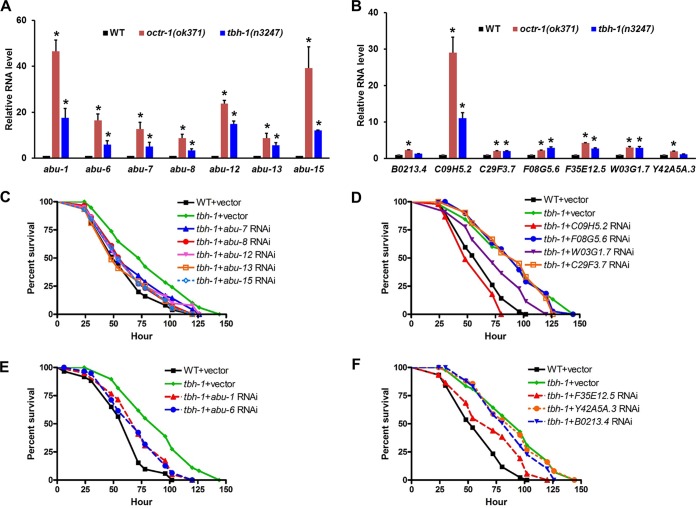
OA functions in OCTR-1-dependent innate immunity. (A) qRT-PCR analysis of *abu* gene expression (*abu-1*, *abu-6*, *abu-7*, *abu-8*, *abu-12*, *abu-13*, and *abu-15*) in *octr-1*(*ok371*) and *tbh-1*(*n3247*) animals relative to wild-type animals exposed to P. aeruginosa. Bars represent means ± SEM. *n* = 3 independent experiments. Asterisks (*) denote a significant difference between the mutant animals and the wild-type animals. (B) qRT-PCR analysis of expression of PMK-1-dependent genes (*B0213.4*, *C09H5.2*, *C29F3.7*, *F08G5.6*, *F35E12.5*, *W03G1.7*, and *Y42A5A.3*) in *octr-1*(*ok371*) and *tbh-1*(*n3247*) animals relative to wild-type animals exposed to P. aeruginosa. Bars represent means ± SEM. *n* = 3 independent experiments. Asterisks (*) denote a significant difference between the mutant animals and the wild-type animals. (C and E) Wild-type and *tbh-1*(*n3247*) animals grown on double-stranded RNA (dsRNA) for vector control or dsRNA for *abu* genes were exposed to P. aeruginosa and scored for survival over time. *P* values represent results of comparisons to *tbh-1* plus vector as follows: WT+vector (*P < *0.0001), *tbh-1*+*abu-7* RNAi (*P = *0.0327), *tbh-1*+*abu-8* RNAi (*P = *0.0039), *tbh-1*+*abu-12* RNAi (*P = *0.0238), *tbh-1*+*abu-13* RNAi (*P = *0.0009), *tbh-1*+*abu-15* RNAi (*P = *0.0019), *tbh-1*+*abu-1* RNAi (*P = *0.0006), *tbh-1*+*abu-6* RNAi (*P = *0.0004). The survival graph is a representative of results from three independent experiments. *n* = 60 animals per strain per experiment. (D and F) Wild-type and *tbh-1*(*n3247*) animals grown on double-stranded RNA (dsRNA) for vector control or dsRNA for *PMK-1-*dependent genes were exposed to P. aeruginosa and scored for survival over time. *P* values represent results of comparisons to *tbh-1*+vector as follows: WT+vector (*P < *0.0001), *tbh-1*+*C09H5.2* RNAi (*P < *0.0001), *tbh-1*+*F08G5.6* RNAi (*P = *0.6207), *tbh-1*+*W03G1.7* RNAi (*P = *0.0033), *tbh-1*+*C29F3.7* RNAi (*P = *0.4595), *tbh-1*+*F35E12.5* RNAi (*P = *0.0011), *tbh-1*+*Y42A5A.3* RNAi (*P = *0.9077), *tbh-1*+*B0213.4* RNAi (*P = *0.2453). The survival graph is a representative of results from three independent experiments. *n* = 45 animals per strain per experiment.

To determine if OA functions in the OCTR-1-dependent pathway, we examined how the lack of OA in *tbh-1*(*n3247*) animals affects the expression of OCTR-1-regulated innate immune genes. OCTR-1 negatively regulates innate immunity by suppressing expression of noncanonical UPR genes of the *pqn* and *abu* (henceforth referred to as *abu*) family and genes in the PMK-1/p38 pathway (12, 13). If OA is a ligand of OCTR-1, these immune genes should be upregulated in *tbh-1*(*n3247*) animals due to the lack of the OCTR-1-dependent suppression, a phenomenon observed in *octr-1*(*ok371*) animals (12, 13). To test this prediction, we used quantitative real-time PCR (qRT-PCR) to measure the expression levels of the immune genes in P. aeruginosa-infected *tbh-1*(*n3247*) animals and compared the levels to those in infected wild-type or *octr-1*(*ok371*) animals. All seven *abu* genes tested (*abu-1*, *abu-6*, *abu-7*, *abu-8*, *abu-12*, *abu-13*, and *abu-15*) and five of the seven PMK-1-dependent genes (*C09H5.2*, *C29F3.7*, *F08G5.6*, *F35E12.5*, *and W03G1.7*) were significantly upregulated in *tbh-1*(*n3247*) animals compared to wild-type animals (Fig. 4A and B), suggesting that OA is part of the OCTR-1 signaling pathway that suppresses the expression of these genes. However, the magnitude of upregulation of most genes in the *tbh-1*(*n3247*) animals was not as high as that observed in the *octr-1*(*ok371*) animals (Fig. 4A and B). These results indicate that an additional pathway(s) that circumvents the necessity of TBH for OA synthesis might exist and produce small amounts of OA that suppress the gene expression. A salvage pathway has been reported for TA synthesis from tyrosine without TDC (25). TBH-independent OA synthesis has not yet been reported. It is also possible that there are OA-independent OCTR-1 pathways, leading to the additional expression of the immune genes in *octr-1*(*ok371*) animals.

To examine whether or not the OCTR-1-regulated innate immune genes are responsible for the improved survival of *tbh-1*(*n3247*) animals, we inactivated these genes by RNA interference (RNAi) in wild-type and *tbh-1*(*n3247*) animals and assayed their survival of P. aeruginosa-mediated killing. RNAi of individual *abu* genes or RNAi of *W03G1.7* or *F35E12.5* partially suppressed the enhanced survival of *tbh-1*(*n3247*) animals (Fig. 4C to F). RNAi of *C09H5.2* fully suppressed the enhanced survival of *tbh-1*(*n3247*) animals (Fig. 4D). Interestingly, this gene was the most highly expressed one in the PMK-1 pathway in *tbh-1*(*n3247*) animals (Fig. 4B). These results illustrate that the elevated immunity of *tbh-1*(*n3247*) animals is due to increased expression of OCTR-1-regulated immune genes, suggesting that OA and OCTR-1 are functionally coupled. In our previous work (12), we performed RNAi of *pmk-1* and *abu* genes in wild-type and *octr-1*(*ok371*) animals and found that their rates of survival were reduced to levels similar to those of *tbh-1*(*n3247*) animals with the same knockdowns (Fig. 2; see also Fig. S8 in reference 12 and Fig. 4 in this study). *octr-1* or *tbh-1* mutations did not confer an additional survival advantage or disadvantage to the animals with the RNAi knockdowns, indicating that these genes function in the same signaling pathway as *pmk-1* or *abu* genes to regulate the nematode’s susceptibility to the pathogen.

### Exogenous administration of OA suppressed OCTR-1-dependent immune responses.

**FIG 5:**
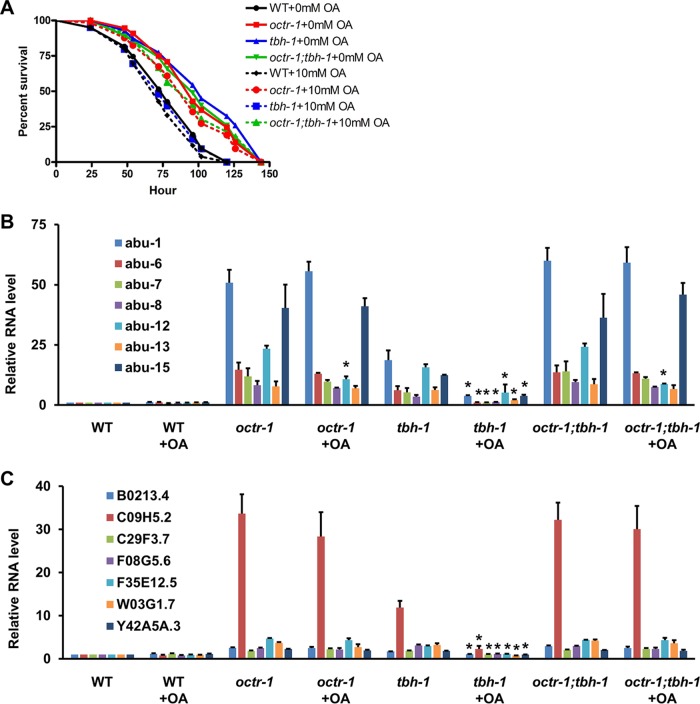
Exogenous OA suppresses OCTR-1-dependent immune responses. (A) Wild-type, *octr-1*(*ok371*), *tbh-1*(*n3247*), and *octr-1*(*ok371*)*;tbh-1*(*n3247*) animals treated with 0 or 10 mM OA were exposed to P. aeruginosa/NGM plates containing 0 or 10 mM OA. The rates of survival of these animals were scored over time. *P* values represent results of comparisons to WT + 10 mM OA animals as follows: *octr-1*(*ok371*)+10 mM OA (*P < *0.0001); *tbh-1*(*n3247*) +10 mM OA (*P = *0.4932); *octr-1*(*ok371*)*;tbh-1*(*n3247*) +10 mM OA (*P < *0.0001). The survival graph is a representative of results from three independent experiments. *n* = 60 animals per strain per experiment. (B) qRT-PCR analysis of *abu* gene expression in untreated or 10 mM OA-treated wild-type, *octr-1*(*ok371*), *tbh-1*(*n3247*), and *octr-1*(*ok371*)*;tbh-1*(*n3247*) animals exposed to P. aeruginosa. Bars represent means ± SEM. *n* = 3 independent experiments. Asterisks (*) denote a significant difference between the same untreated and OA-treated strains. (C) qRT-PCR analysis of the expression of the PMK-1-dependent genes in untreated or 10 mM OA-treated wild-type, *octr-1*(*ok371*), *tbh-1*(*n3247*), and *octr-1*(*ok371*)*;tbh-1*(*n3247*) animals exposed to P. aeruginosa. Bars represent means ± SEM. *n* = 3 independent experiments. Asterisks (*) denote a significant difference between the same untreated and OA-treated strains.

Exogenous administration of putative ligands has been used to study ligand-receptor coupled responses in various organisms, including C. elegans (23, 25, 33). Because TBH deficiency in *tbh-1*(*n3247*) animals greatly reduces or completely abolishes OA production, we expected that addition of exogenous OA to these animals would rescue their mutant phenotype. OA was serially diluted to 0 to 100 mM in S basal buffer and incubated with wild-type, *octr-1*(*ok371*), *tbh-1*(*n3247*), and *octr-1*(*ok371*)*;tbh-1*(*n3247*) animals for 1 h (23). After incubation, the animals were exposed to P. aeruginosa for survival assays, and levels of expression of *abu* genes and the PMK-1-dependent genes were measured by qRT-PCR. The 10 to 25 mM doses of OA rescued the *erp* of *tbh-1*(*n3247*) animals with respect to P. aeruginosa (Fig. 5A; see also Fig. S2A), confirming that OA is involved in C. elegans immunity. Rescue was not observed in the *octr-1*(*ok371*)*;tbh-1*(*n3247*) double mutants (Fig. 5A; see also Fig. S2A), indicating that the function of OA in immunity depends on the presence of OCTR-1. Of note, OA at 4 mM had no effect on the survival of *tbh-1*(*n3247*) animals against P. aeruginosa (Fig. S2B), while OA at 50 mM or higher concentrations inhibited locomotion of both wild-type and mutant animals (data not shown). The physiological OA content in an adult worm was estimated to be about 3 µM (i.e., 5 ± 2 pmol per mg of wet weight [34, 35], assuming that an adult worm weights about 4 µg [36], with a body volume of 5.81 nl [37]). The concentrations of exogenous OA that we used in the experiments described above were much higher than the physiological OA concentration. It is not clear how much exogenous OA was taken up or metabolized by the worms, but that portion is expected to be very small due to the low permeability of the C. elegans cuticle with respect to small molecules (38). Pharmacological studies showed that drug concentrations effective on C. elegans are often several orders of magnitude higher than those used for cultured cells (39), which is in support of the idea that the low permeability of the C. elegans cuticle restricts drug uptake.

10.1128/mBio.01645-18.3 FIG S2 Effects of 25 mM or 4 mM exogenous OA on OCTR-1-dependent immune responses. Download FIG S2, TIF file, 0.4 MB. Copyright © 2018 Sellegounder et al. 2018 Sellegounder et al. This content is distributed under the terms of the Creative Commons Attribution 4.0 International license.

Consistent with the survival assays described above, 10 mM exogenous OA significantly decreased the expression of *abu* genes (Fig. 5B) and the PMK-1-dependent genes (Fig. 5C) in P. aeruginosa-infected *tbh-1*(*n3247*) animals, but such decreases were not observed in infected *octr-1*(*ok371*) animals or *octr-1*(*ok371*)*;tbh-1*(*n3247*) double mutants (Fig. 5B and C), except the *abu-12* gene, which was downregulated by OA in all mutant strains. These results suggest that the animals absorbed exogenous OA which then functioned through OCTR-1 to suppress innate immune responses to P. aeruginosa.

It has been reported that OA can induce a starvation-like response (40–43) which could potentially affect the nematode’s survival against P. aeruginosa. To test this possibility, we induced a starvation response in wild-type animals by placing young-adult animals onto nematode growth medium (NGM) plates without bacterial food for 6 h (41) and then transferred them to a P. aeruginosa lawn for survival assays. The starved animals displayed a level of susceptibility to P. aeruginosa infection similar to that seen with well-fed control animals (Fig. S3), indicating that a starvation response does not contribute to the nematode’s survival against P. aeruginosa. This result is consistent with our conclusion that OA functions via immune regulation to impact the animals’ survival.

10.1128/mBio.01645-18.4 FIG S3 Starvation does not alter C. elegans survival of P. aeruginosa challenge. Download FIG S3, TIF file, 0.2 MB. Copyright © 2018 Sellegounder et al. 2018 Sellegounder et al. This content is distributed under the terms of the Creative Commons Attribution 4.0 International license.

**FIG 6:**
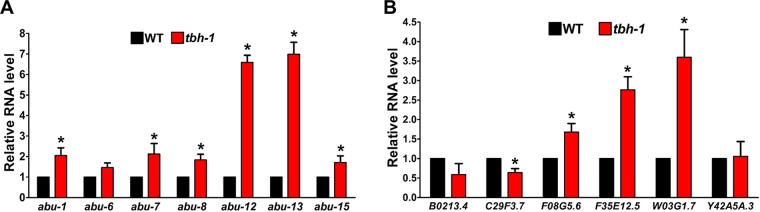
Basal expression of *abu* genes and PMK-1-dependent genes in wild-type and *tbh-1*(*n3247*) animals. Data represent results of qRT-PCR analysis of the expression of *abu* genes (A) and PMK-1-dependent genes (B) in *tbh-1*(*n3247*) animals relative to wild-type animals. Bars represent means ± SEM. *n* = 3 independent experiments. Asterisks (*) denote a significant difference between the mutant animals and wild-type animals.

We also examined the basal levels of expression of *abu* genes and the PMK-1-dependent genes in wild-type and *tbh-1*(*n3247*) animals and investigated how exogenous administration of OA affects these basal levels of expression. The mutant animals displayed significantly higher basal expression of six of the seven *abu* genes tested and three of the six PMK-1-dependent genes tested than the wild-type animals (Fig. 6). This observation supports the idea of the existence of an octopaminergic immunoinhibitory pathway in wild-type animals under basal or normal conditions. Addition of 10 mM exogenous OA did not significantly change the level of expression of most of these genes in wild-type animals (Fig. S4). A plausible explanation would be that the endogenous OA level in wild-type animals is high enough for mediating octopaminergic immune regulation and that addition of more OA would not further enhance this signaling pathway. In *tbh-1*(*n3247*) animals, however, most *abu* genes and the PMK-1-dependent genes were downregulated by exogenous OA (Fig. S5), indicating restoration of the octopaminergic immunoinhibitory signaling in the mutants. These results are consistent with our model that an octopaminergic immunoinhibitory pathway is tonically active in wild-type animals under normal conditions to maintain immunological homeostasis, probably to suppress unwanted innate immune responses.

10.1128/mBio.01645-18.5 FIG S4 qRT-PCR analysis of the expression of *abu* genes and PMK-1-dependent genes in wild-type animals with 10 mM OA treatment relative to no treatment. Download FIG S4, TIF file, 0.2 MB. Copyright © 2018 Sellegounder et al. 2018 Sellegounder et al. This content is distributed under the terms of the Creative Commons Attribution 4.0 International license.

10.1128/mBio.01645-18.6 FIG S5 qRT-PCR analysis of the expression of *abu* genes and PMK-1-dependent genes in *tbh-1*(*n3247*) animals with 10 mM OA treatment relative to no treatment. Download FIG S5, TIF file, 0.2 MB. Copyright © 2018 Sellegounder et al. 2018 Sellegounder et al. This content is distributed under the terms of the Creative Commons Attribution 4.0 International license.

### OA is an endogenous ligand of OCTR-1 in immunity regulation.

**FIG 7:**
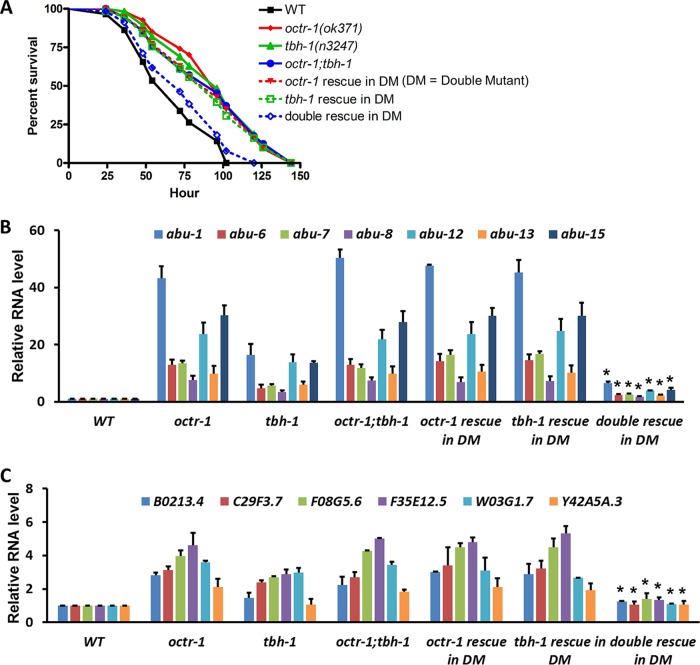
OA acts as an endogenous ligand of OCTR-1 in immunity regulation. (A) Wild-type and mutant animals were exposed to P. aeruginosa and scored for survival over time. DM, double mutant *octr-1*(*ok371*)*;tbh-1*(*n3247*). *P* values represent results of comparisons to WT animals as follows: *octr-1*(*ok371*) (*P < *0.0001); *tbh-1*(*n3247*) (*P < *0.0001); *octr-1*(*ok371*)*;tbh-1*(*n3247*) (*P < *0.0001); TBH-1 rescue in DM (*P < *0.0001); OCTR-1 rescue in DM (*P < *0.0001); TBH-1 and OCTR-1 double rescue in DM (*P = *0.1443). The survival graph is a representative of results from three independent experiments. *n* = 60 animals per strain per experiment. (B) qRT-PCR analysis of *abu* gene expression in mutant animals relative to wild-type animals exposed to P. aeruginosa. Bars represent means ± SEM. *n* = 3 independent experiments. Asterisks (*) denote a significant difference between the double mutant animals and the double rescue animals. (C) qRT-PCR analysis of the expression of the PMK-1-dependent genes in mutant animals relative to wild-type animals exposed to P. aeruginosa. Bars represent means ± SEM. *n* = 3 independent experiments. Asterisks (*) denote a significant difference between the double mutant animals and the double rescue animals. Expression of *C09H5.2* is shown in Fig. S7 in the supplemental material.

The results of the experiments performed with exogenous OA as described above indicate that OA acts as a ligand of OCTR-1 in immunity regulation. To determine if OA is an endogenous ligand of OCTR-1, we generated three rescue strains to restore OA production and/or OCTR-1 expression in *octr-1*(*ok371*)*;tbh-1*(*n3247*) double mutant animals (Table 1). The function of endogenous OA in the presence or absence of OCTR-1 was tested by survival assays and by measurements of the expression of *abu* genes and the PMK-1-dependent genes. Restoration of TBH or OCTR-1 expression alone in *octr-1*(*ok371*)*;tbh-1*(*n3247*) animals did not rescue their *erp* with respect to P. aeruginosa, but restoration of both TBH and OCTR-1 did (Fig. 7A). In agreement with the survival assays, the expression levels of the immune genes did not change significantly in *octr-1*(*ok371*)*;tbh-1*(*n3247*) animals with restoration of either TBH or OCTR-1 but were significantly reduced with the restoration of both TBH and OCTR-1 (Fig. 7B and C; see also Fig. S6). These data suggest that OA functions as an endogenous ligand of OCTR-1 to regulate the innate immune response to P. aeruginosa infection.

**TABLE 1:** Rescue strains of *octr-1*(*ok371*)*;tbh-1*(*n3247*) animals

Strain	Genetic background
TBH rescue	*octr-1*(*ok371*)*;tbh-1*(*n3247*)*;tbh-1p*::*tbh-1*::*mCherry*
OCTR-1 rescue	*octr-1*(*ok371*)*;tbh-1*(*n3247*)*; octr-1p*::*octr-1*::*gfp*
TBH and OCTR-1 rescue	*tbh-1*(*n3247*)*;tbh-1p*::*tbh-1*::*mCherry;octr-1*(*ok371*)*;octr-1p*::*octr-1*::*gfp*

10.1128/mBio.01645-18.7 FIG S6 qRT-PCR analysis of the expression of PMK-1-dependent gene *C09H5.2* in mutant animals relative to wild-type animals exposed to P. aeruginosa. Download FIG S6, TIF file, 0.2 MB. Copyright © 2018 Sellegounder et al. 2018 Sellegounder et al. This content is distributed under the terms of the Creative Commons Attribution 4.0 International license.

10.1128/mBio.01645-18.8 FIG S7 qRT-PCR analysis of *lips-6* expression in wild-type, *tbh-1;octr-1* double mutant, *tbh-1* rescue, and double rescue animals in the well-fed or the fasted state. Download FIG S7, TIF file, 0.2 MB. Copyright © 2018 Sellegounder et al. 2018 Sellegounder et al. This content is distributed under the terms of the Creative Commons Attribution 4.0 International license.

To additionally confirm the functionality of OA in the *tbh-1* rescue strains besides using survival assays and measurements of immune gene expression, we performed qRT-PCR to quantify the expression of lipase gene *lips-6* in wild-type animals, *tbh-1;octr-1* double mutants, and *tbh-1* rescue and double rescue animals. OA or starvation-induced OA was implicated in promoting *lips-6* expression, which elicits lipid mobilization (40). We observed that for animals in the well-fed state, *lips-6* expression was higher in the double mutants than in the wild-type animals and the two rescue strains despite the double mutants’ lack of OA, likely reflecting a higher basal level of lipid metabolism in the double mutants (Fig. S7). In animals in the fasted state, however, *lips-6* expression was 4-fold to 5-fold higher in the wild-type animals and in the two rescue strains than in the double mutants (Fig. S7), indicating that restoration of OA production in the rescue strains mediates starvation-triggered lipid hydrolysis. Of note, *tbh-1* rescue in double mutants was able to induce *lips-6* expression in the fasted state without the presence of OCTR-1 (Fig. S7), suggesting that OCTR-1 is not involved in OA-dependent lipid metabolism. This is in agreement with the findings by Tao et al. (40) indicating that OA functions through another GPCR, SER-3, to mediate lipid hydrolysis.

### OA-producing RIC neurons function in OCTR-1-dependent neural regulation of innate immunity.

**FIG 8:**
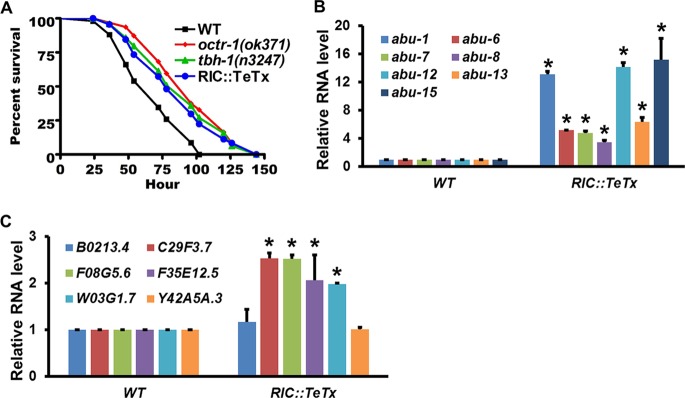
RIC neurons function in OCTR-1-dependent innate immunity. (A) Wild-type and mutant animals were exposed to P. aeruginosa and scored for survival over time. *P* values represent results of comparisons to WT animals as follows: *octr-1*(*ok371*) (*P < *0.0001); *tbh-1*(*n3247*) (*P < *0.0001); RIC::TeTx (*P < *0.0001). The survival graph is a representative of results from three independent experiments. *n* = 60 animals per strain per experiment. (B) qRT-PCR analysis of *abu* gene expression in RIC::TeTx animals relative to wild-type animals exposed to P. aeruginosa. Bars represent means ± SEM. *n* = 3 independent experiments. Asterisks (*) denote a significant difference between RIC::TeTx animals and WT animals. (C) qRT-PCR analysis of the expression of PMK-1-dependent genes in RIC::TeTx animals relative to wild-type animals exposed to P. aeruginosa. Bars represent means ± SEM. *n* = 3 independent experiments. Asterisks (*) denote a significant difference between RIC::TeTx animals and WT animals. Expression of *C09H5.2* is shown in Fig. S8.

In C. elegans, OA is synthesized in two RIC interneurons and in nonneuronal gonadal sheath cells (34). To determine if RICs are involved in the OCTR-1 neural circuit, we examined how blocking synaptic transmission in RIC neurons affects the nematode’s survival of P. aeruginosa challenge and the expression of OCTR-1-regulated innate immune genes. We generated transgenic RIC::TeTx (*tbh-1p*::*TeTx*::*SL2*::*GFP*) animals in which RIC neuronal activity was blocked by tetanus toxin (TeTx). TeTx is a potent clostridial neurotoxin; expression of the light chain of TeTx blocks synaptic transmission via cleaving the synaptic vesicle protein synaptobrevin (44, 45). Similarly to *octr-1*(*ok371*) or *tbh-1*(*n3247*) animals, RIC::TeTx animals showed enhanced resistance to P. aeruginosa-mediated killing compared to wild-type animals (Fig. 8A). qRT-PCR measurements show that *abu* genes and the PMK-1-dependent genes were significantly upregulated in RIC::TeTx animals relative to wild-type animals exposed to P. aeruginosa (Fig. 8B and C; see also Fig. S8). These results demonstrate that blocking synaptic transmission in RICs leads to *erp* with respect to P. aeruginosa and upregulation of OCTR-1-regulated immune genes, indicating that the function of RICs in the OCTR-1 circuit is to negatively regulate innate immunity.

10.1128/mBio.01645-18.9 FIG S8 qRT-PCR analysis of the expression of PMK-1-dependent gene *C09H5.2* in wild-type and RIC::TeTx animals exposed to P. aeruginosa. Download FIG S8, TIF file, 0.1 MB. Copyright © 2018 Sellegounder et al. 2018 Sellegounder et al. This content is distributed under the terms of the Creative Commons Attribution 4.0 International license.

**FIG 9:**
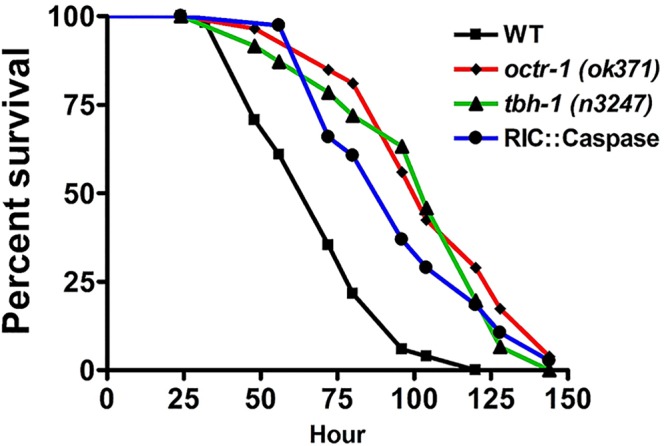
RIC ablation enhances C. elegans resistance to P. aeruginosa. Wild-type and RIC-ablated animals (RIC::Caspase) were exposed to P. aeruginosa and scored for survival over time. *P* values represent results of comparisons to WT animals as follows: *octr-1*(*ok371*) (*P < *0.0001); *tbh-1*(*n3247*) (*P < *0.0001); RIC::Caspase (*P < *0.0001). The survival graph is a representative of results from three independent experiments. *n* = 45 to 60 animals per strain per experiment.

To confirm the role of RICs in immune regulation, we also genetically ablated RICs using the recCaspase system (46). RICs were selectively targeted for elimination by the combinational use of the *cat-1* and *tbh-1* promoters that drive the expression of two caspase subunits overlapping only in RIC neurons. The resulting RIC-ablated animals (strain JRS9; see Table S1 in the supplemental material) were subsequently examined for survival of P. aeruginosa-mediated killing. Similarly to *octr-1*(*ok371*) or *tbh-1*(*n3247*) animals, RIC-ablated animals showed enhanced resistance to P. aeruginosa compared to wild-type animals (Fig. 9), indicating that RICs negatively modulate innate immunity. This result is consistent with the finding of the experiment performed as described above with RIC::TeTx animals, i.e., that the function of RICs in the OCTR-1 circuit is to suppress the innate immune response.

10.1128/mBio.01645-18.10 TABLE S1 List of transgenic strains generated in this study. Download Table S1, PDF file, 0.1 MB. Copyright © 2018 Sellegounder et al. 2018 Sellegounder et al. This content is distributed under the terms of the Creative Commons Attribution 4.0 International license.

### RIC neurons are deactivated by P. aeruginosa PA14 but activated by E. coli OP50.

**FIG 10:**
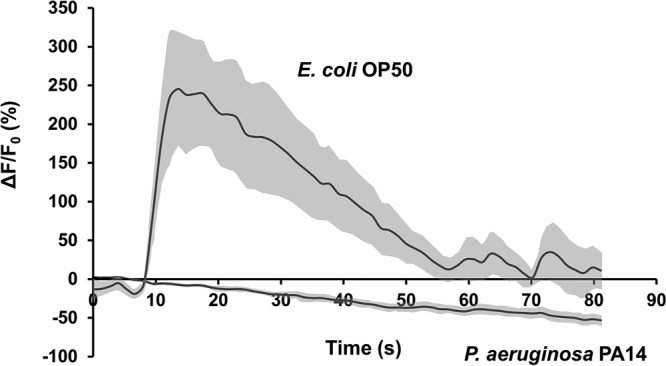
Neuronal activity of RICs was measured by calcium imaging. RIC neurons in transgenic *tbh-1p*::*GCaMP6s* animals respond to exposure to E. coli OP50 or P. aeruginosa PA14. The trace represents averages of GCaMP fluorescence changes. The light-gray shading around the trace indicates SEM. Base line, average GCaMP fluorescence from 0 to 9 s with flow of LB broth. ΔF/F_0_ (%), percentage fluorescence change. *n* = 9 neurons.

The studies described above indicate the existence of an octopaminergic immunoinhibitory pathway in C. elegans. To discover how this pathway is regulated, we employed calcium imaging to monitor RIC activity under infectious conditions (i.e., exposure to P. aeruginosa strain PA14) or normal conditions (i.e., exposure to standard worm food [E. coli strain OP50]). Our experiments were conducted with a microfluidic device that restrains the worm with the tip of the head (where chemosensory cilia are located) in a fluidic stream that can be rapidly switched between different stimuli (47). By specifically expressing a fluorescence-based calcium indicator (GCaMP6s) in RICs under the control of a *tbh-1* promoter, we observed that exposing the *tbh-1p*::*GCaMP6s* transgenic animals to P. aeruginosa caused a sustained calcium decrease (Fig. 10), indicating that RICs are gradually deactivated in the presence of the pathogen. In contrast, exposing the transgenic animals to nonpathogenic E. coli OP50 transiently increased calcium flux in RIC neurons (Fig. 10), suggesting that RICs are activated by these bacteria. The results described above indicate that exposure of C. elegans to E. coli OP50 activates the octopaminergic immunoinhibitory pathway, probably to suppress unwanted innate immune responses, whereas P. aeruginosa exposure downregulates the octopaminergic immunoinhibitory pathway to allow enhanced innate immune responses.

## DISCUSSION

**FIG 11:**
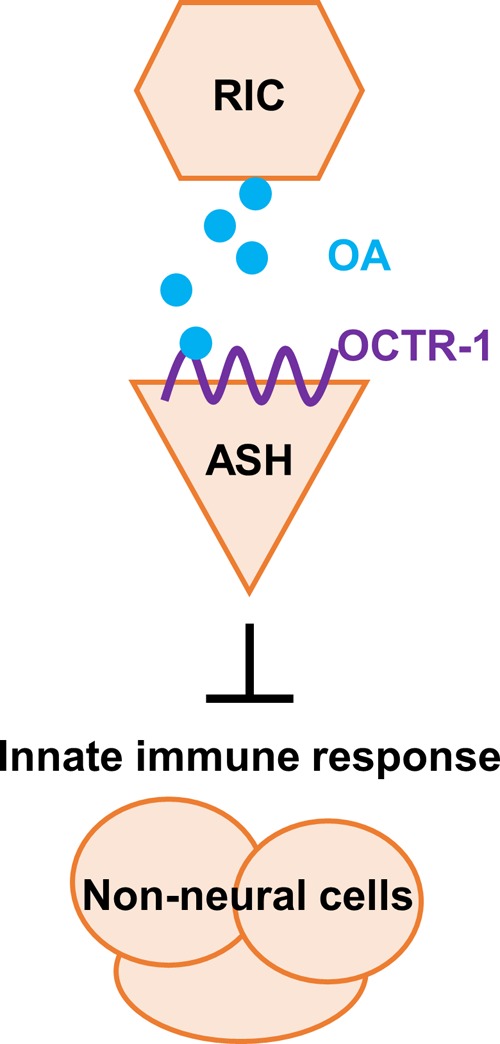
The octopaminergic immunoinhibitory pathway in C. elegans. In this pathway, OA released from RIC neurons acts as a ligand of OCTR-1 that functions in the sensory ASH neurons to suppress innate immune responses in nonneural tissues.

In this study, we uncovered an octopaminergic immunoinhibitory pathway in C. elegans: OA released from RIC neurons acts as a ligand of OCTR-1 that functions in the sensory ASH neurons to suppress innate immune responses in pharyngeal and intestinal tissues (Fig. 11) (12, 13). Inactivation of this pathway leads to enhanced innate immune responses to bacterial infection. OA is the invertebrate counterpart of the vertebrate neurotransmitter norepinephrine (25, 48). Both neurotransmitters modulate multiple physiological and behavioral processes by binding to GPCRs. The OCTR-1 OA receptor is orthologous to the alpha-2A adrenergic receptor of norepinephrine (77.9% sequence homology; Wormbase). Since norepinephrine regulates innate immune responses in mammals and other vertebrates (49–53), the immunomodulatory function of OA or norepinephrine is evolutionarily conserved. Sympathetic noradrenergic neurons, especially those of the vagally innervated splenic nerve (52), function in the mammalian inflammatory reflex to suppress infection-induced production of inflammatory cytokines in the spleen and other organs (54, 55). Analogously to what RIC neurons do in C. elegans, these noradrenergic neurons modulate the innate immune response in mammals. Despite the advancements in mammal studies, details about the functions of individual neurons and neural circuits in immunomodulation are lacking due to the difficulty encountered in efforts to dissect the highly complex mammalian nervous system. By using C. elegans in the current study, we revealed specific neurons and molecules involved in this process. Our study results support a model whereby an octopaminergic immunoinhibitory neural circuit is tonically active under normal conditions and can be deactivated under pathogen infection or abnormal physiological conditions. This model is consistent with clinical evidence indicating that decreased or severely impaired anti-inflammatory neural circuits represent the major underlying causes for many innate immune diseases such as sepsis, arthritis, inflammatory bowel disease, and hemorrhagic shock (54). Recent preclinical studies have been focused on stimulating the neural circuits electrically or pharmacologically (54). Elucidating octopaminergic regulation of innate immunity could be helpful to the development of new treatments for these innate immune diseases.

In the experiments described above where we restored TBH and/or OCTR-1 expression in *octr-1*(*ok371*)*;tbh-1*(*n3247*) double mutant animals (Table 1) to determine if OA is an endogenous ligand of OCTR-1, we observed that expression of *C09H5.2* (one of the PMK-1-dependent genes) was upregulated more in double mutant animals than in either *octr-1* or *tbh-1* single mutants (see Fig. S6 in the supplemental material), indicating that both OCTR-1 and OA mediate additional suppression of *C09H5.2* expression outside the OA-OCTR-1 pathway. Although the *C09H5.2* expression level in *octr-1* mutant animals resembles that in *octr-1*-rescue animals (Fig. S6), *C09H5.2* was upregulated through different mechanisms in these two types of animals. In *octr-1* mutant animals, *C09H5.2* upregulation is mediated through a total lack of OCTR-1-mediated suppression; in *octr-1*-rescue animals, such upregulation is mediated through a total lack of OA-mediated suppression plus a partial lack of OCTR-mediated suppression, depending on the expression level of rescued OCTR-1. Because of this difference, *C09H5.2* levels of expression in the rescued animals and *octr-1* mutant animals have little correlation.

Multiple neurotransmitters modulate innate immunity in C. elegans. For instance, Anderson et al. reported that during infection with *M. nematophilum*, signaling by sensory neurons via neurotransmitter serotonin can suppress the immune response in the rectal epithelium and reductions in serotonin synthesis result in an increased immune response (16). In comparison, during P. aeruginosa infection, loss of serotonin has no effect on cellular immunity (56). Instead, we found that OA modulates P. aeruginosa-triggered immune responses by suppressing OCTR-1-regulated innate immune genes such as the *abu* genes and genes in the PMK-1 pathway. *abu* genes encode prion-like glutamine- and asparagine-rich (PQN) proteins that are components of the pharyngeal cuticle in C. elegans (57). The cuticular matrix forms the first barrier to infection; this defense mechanism of ABU proteins could work in parallel with their immune function (12, 13, 58, 59) in the nematode’s overall defense against pathogen infection. The C. elegans genome contains three OA GPCRs, namely, OCTR-1, SER-6, and SER-3 (23, 60, 61). In *octr-1*(*ok371*)*;tbh-1*(*n3247*) double mutants, restoration of OA production was unable to rescue the function of OA in immunity (Fig. 7A), indicating that OCTR-1 is the only GPCR that OA uses to mediate P. aeruginosa-triggered immune responses. On the other hand, OA is possibly not the only ligand for the OCTR-1 pathway, because P. aeruginosa infection induced significantly higher upregulation of immune genes in *octr-1*(*ok371*) animals than in *tbh-1*(*n3247*) animals (Fig. 4A and B), indicating that OA-independent OCTR-1 pathways might also exist, leading to the additional expression of immune genes in *octr-1*(*ok371*) animals. Very recently, Cao and Aballay reported that dopamine originating in CEP neurons inhibits innate immune responses to P. aeruginosa through a D1-like dopamine receptor (DOP-4) in ASG neurons (17). It appears that multiple neurotransmitters can modulate immune responses to the same pathogens.

## MATERIALS AND METHODS

### Supplemental methods.

Supplemental information about the methods used in this study is presented in Text S1 in the supplemental material.

10.1128/mBio.01645-18.1 TEXT S1 Supplemental Methods. The following methods are included in Supplemental Methods: C. elegans survival assay, pharyngeal pumping rate assay, defecation rate assay, bacterial lawn avoidance assay, profile of bacterial accumulation in the nematode intestine, quantification of intestinal bacterial loads, RNA interference, RNA isolation, quantitative real-time PCR, and starvation of worms. Download Text S1, PDF file, 0.1 MB. Copyright © 2018 Sellegounder et al. 2018 Sellegounder et al. This content is distributed under the terms of the Creative Commons Attribution 4.0 International license.

### C. elegans and bacterial strains.

The wild-type animal strain was C. elegans Bristol N2. *octr-1*(*ok371*), *tbh-1*(*n3247*), and *tdc-1*(*n3419*) strains were obtained from the Caenorhabditis elegans Genetics Center (University of Minnesota, Minneapolis, MN). Double mutant strain *octr-1*(*ok371*)*;tbh-1*(*n3247*), rescue strains, and other transgenic animals were constructed using standard genetic techniques as described below. C. elegans strains were cultured under standard conditions (62). E. coli strain OP50 and P. aeruginosa strain PA14 (6) were grown in Luria-Bertani (LB) broth at 37°C.

### Plasmid construction and transgenic-animal generation.

A *tbh-1* genomic DNA fragment (3,030 bp) was amplified by PCR from wild-type C. elegans at mixed stages using oligonucleotides 5′-AAAGCTAGCATGAGAAGTGCCGTTGCTCT-3′ and 5′-CCCGGTACCTTACTCAAAATTAATCATGTCATTGATGGC-3′. The pCY011G plasmid (*tbh-1p*::*tbh-1g*::*sl2*::*gfp*) was constructed by cloning this *tbh-1*g into plasmid pXJ30 via the NheI and KpnI sties to replace *tdc-1* cDNA. The resulting construct, pCY011G, was microinjected into *octr-1*(*ok371*)*;tbh-1*(*n3247*) animals at 25 ng/µl with *unc-122p*::*gfp* (10 ng/µl) as a coinjection marker to generate strain JRS2.

The *octr-1* promoter (3,957 bp) was amplified by PCR from the genomic DNA of wild-type C. elegans at mixed stages using oligonucleotides 5′-CACTGCAGAGTTTTTTCCACCAATATTTTCCGCTT-3′ and 5′-CCGGGATCCGAAAATGTGAAGTGTTGGTAGA-3′ and was cloned into vector pPD95.77 (Fire Lab C. elegans vector kit; Addgene, Cambridge, MA) via the PstI and BamHI sties. A *octr-1* cDNA fragment (1,227 bp) was amplified by PCR from a cDNA pool of wild-type C. elegans at mixed stages using oligonucleotides 5′-AAAGGATCCATGTGGAACCTTAACTGCAGTGA-3′ and 5′-ATCGGATCCTCATTTGTAGAACTCCATGAGTGGATG-3′ and was cloned into vector pPD95.77 containing *octr-1p* via BamHI site to create plasmid pCY021 (*octr-1p*::*octr-1c*::*sl2*::*gfp*). This plasmid was microinjected into *octr-1*(*ok371*)*;tbh-1*(*n3247*) animals at 40 ng/µl with *myo-2p*::*DsRed* (10 ng/µl) as a coinjection marker to generate strain JRS4.

Plasmid pCY011M (*tbh-1p*::*tbh-1g*::*sl2*::*mCherry*) was constructed by using plasmid pNP502 as the backbone. A *tbh-1* promoter sliced from vector pXJ30 by the use of FseI and AscI replaced the *tdc-1* promoter in pNP502. The 3,030 bp of *tbh-1*g sliced from pCY011G by NheI and KpnI replaced HisCl cDNA in pNP502. Plasmids pCY011M and pCY021 were microinjected into *octr-1*(*ok371*)*;tbh-1*(*n3247*) animals at 25 ng/µl and 40 ng/µl, respectively, to generate strain JRS5. Two coinjection markers, *unc-122p*::*gfp* (10 ng/µl) and *myo-2p*::*DsRed* (10 ng/µl), were used.

Plasmid pDS01 (*tbh-1p*::*GCaMP6s*) was constructed by cloning 4,556 bp of the *tbh-1* promoter from pXJ30 and replaced the *rgef-1* promoter between the HindIII and BamHI sites in pCB101. The resulting construct, pDS01, was microinjected into wild-type animals at 22 ng/µl with *unc-122p*::*gfp* (10 ng/µl) as a coinjection marker to generate strain JRS6 for RIC neuron calcium imaging.

Plasmid pCY01T (*tbh-1p*::*TeTx*::*mCherry*) was created by using pNP302 as the backbone. A *tbh-1* promoter sliced from pXJ30 by the use of FseI and AscI replaced the *tdc-1* promoter in pNP302. The resulting construct, pCY01T, was microinjected into wild-type animals at 50 ng/µl with *unc-122p*::*gfp* (10 ng/µl) as a coinjection marker to generate strain JRS8.

All transgenic strains constructed in this study are listed in Table S1 in the supplemental material. The sequences of all genes and promoters were verified by sequencing at Eton Bioscience (Research Triangle Park, NC). The expression patterns of neuron-specific promoters were confirmed for the relevant transgenic strains. Plasmids pXJ30, pNP502, pXJ08, and pNP302 were gifts from Cori Bargmann at Rockefeller University. Plasmid pCB101 was a gift from Aravinthan Samuel at Harvard University.

### Octopamine administration.

Wild-type, *octr-1*(*ok371*), *tbh-1*(*n3247*), and *octr-1*(*ok371*)*;tbh-1*(*n3247*) gravid adult animals were transferred to NGM plates containing E. coli OP50 and allowed to lay eggs for 1 h. The gravid adults were removed, and the eggs were allowed to develop at 20°C to reach the young-adult stage. The synchronized young-adult animals were collected and soaked in S basal buffer containing 0 mM, 4 mM, 10 mM, 25 mM, 50 mM, or 100 mM octopamine (Fisher Scientific) with shaking for 1 h at room temperature before subsequent assays were performed. To prepare the bacterial lawns used for C. elegans survival assays, a 10-ml overnight culture of P. aeruginosa PA14 was centrifuged at 4,000 rpm for 10 min. The bacterial pellet was resuspended in 1 ml P. aeruginosa supernatant to make a 10× concentrated cell culture. A 50-μl volume of the 10× cell culture was placed on a 3.5-cm-diameter modified NGM agar plate containing octopamine at the concentrations assayed. Octopamine-soaked animals were transferred to these NGM plates containing P. aeruginosa PA14 and were then used in survival assays. To prepare bacterial lawns for C. elegans qRT-PCR experiments, a 100-ml overnight culture of P. aeruginosa PA14 was centrifuged at 4,000 rpm for 10 min. The bacterial pellet was resuspended in 10 ml P. aeruginosa supernatant to make a 10× concentrated cell culture. A 200-μl volume of 10× cell culture was placed on a 10-cm-diameter modified NGM agar plate containing octopamine at the concentrations assayed. Octopamine-soaked animals were transferred to these NGM plates containing P. aeruginosa PA14 and incubated for 4 h at 25°C. The animals were then collected and used for RNA isolation and qRT-PCR experiments.

### Genetic ablation of RIC neurons.

RICs were genetically ablated using the recCaspase system, which targets specific cells for apoptosis by coexpression of the two subunits of caspase CED-3 under the control of overlapping promoters (46). RICs were targeted by the combinational use of *cat-1* and *tbh-1* promoters. These promoters were cloned into plasmids TU#813 and TU#814 (gifts from Martin Chalfie; Addgene plasmids no. 16082 and 16083), resulting in plasmids pDS02 and pDS03, respectively, which drive the expression of the individual subunits of CED-3 in RICs. Specifically, pDS02 [*cat-1p*::*Caspase 3*(*p12*)::*nz*] was generated by replacing *Pmec-18* in TU#813 with a 2-kb *cat-1* promoter amplified by PCR from genomic DNA of wild-type C. elegans at mixed stages using oligonucleotides 5′-TATTCGCATGCACACGCACATTGGCACTT-3′ and 5′-CTGGTGGATCCACCTCCTTCTTCCAAGTT-3′. Cloning was done by restriction digestion of the PCR products and TU#813 with SphI and BamHI, followed by ligation of the digested fragments. Similarly, pDS03 [*tbh-1p*::*cz*::*Caspase* (*p17*)] was constructed by replacing *Pmec-18* in TU#814 with a 4.5-kb *thb-1* promoter obtained from pDS01. Cloning was done by restriction digestion of plasmids TU#814 and pDS01 with HindIII and BamHI, followed by ligation of the digested fragments. Plasmids pDS02 and pDS03 were microinjected at concentrations of 60 ng/µl and 40 ng/µl, respectively, into strain MT9971 {*nIs107* [*tbh-1*::*GFP* + *lin-15*(+)] *III*]} along with coinjection marker *coel*::*DsRed* (10 ng/µl) to generate strain JRS9 (Table S1). RIC ablation was confirmed by the loss of *gfp* expression and the presence of coinjection marker *coel*::*DsRed*. Three independent positive-testing lines were selected and maintained for further assays.

### Calcium imaging.

Transgenic C. elegans *tbh-1p*::*GCaMP6s* animals were grown to the young-adult stage. Individual animals were placed in a custom-designed poly-di-methyl-siloxane (PDMS) microfluidic device that restrains the worm with the tip of the head (where the chemosensory cilia are located) in a fluidic stream that can be rapidly switched to different stimuli (47). Fluorescence from RIC neurons was captured using a Leica confocal compound microscope (model DMi8) equipped with a scientific complementary metal-oxide semiconductor (sCMOS) camera. We first captured the baseline activity of RIC neurons in LB buffer and then exposed the animals to E. coli OP50 or P. aeruginosa PA14. High-resolution images were recorded at a rate of 0.74 frames/s. Image series obtained from each experiment were analyzed using ImageJ software. Changes in fluorescence intensity were quantified manually for defined regions of interest (ROI), and background fluorescence was corrected for each experiment using the same ROI data. For RIC neurons, changes in GCaMP6s were mostly dominant in the axon region. Hence, we measured the change in fluorescence defining ROI for the axon region. Images from individual experiment were registered using the stackreg option available with ImageJ. To analyze the responses to a stimulus (E. coli OP50 or P. aeruginosa PA14), the average fluorescence and standard error were calculated by setting 0 to 9 s as the baseline for LB. The average fluorescence of LB was set as *F*_0_. Percentage changes in fluorescence [(*F* − *F*_0_)/*F*_0_] were plotted against time in the combined time windows.

### Statistical analysis.

For C. elegans survival assays, animal survival data were plotted as a nonlinear regression curve using PRISM software (version 6, GraphPad Software, Inc. La Jolla, CA). Survival curves were considered significantly different from the curves determined from the appropriate controls indicated in the main text for *P* values of <0.05. Prism uses the product limit or Kaplan-Meier method to calculate survival fractions and the log rank test (equivalent to the Mantel-Haenszel test) to compare survival curves. A two-sample *t* test for independent samples was used to analyze qRT-PCR results; *P* values of <0.05 were considered significant. All the experiments were repeated at least 3 times except where otherwise indicated.
