# Supplementary figures and images for: Octopaminergic Signaling Mediates Neural Regulation of Innate Immunity in Caenorhabditis elegans
Source: mBio. 2018 Oct 9;9(5):e01645-18. doi: 10.1128/mBio.01645-18 (PMC6178624; doi:10.1128/mBio.01645-18)

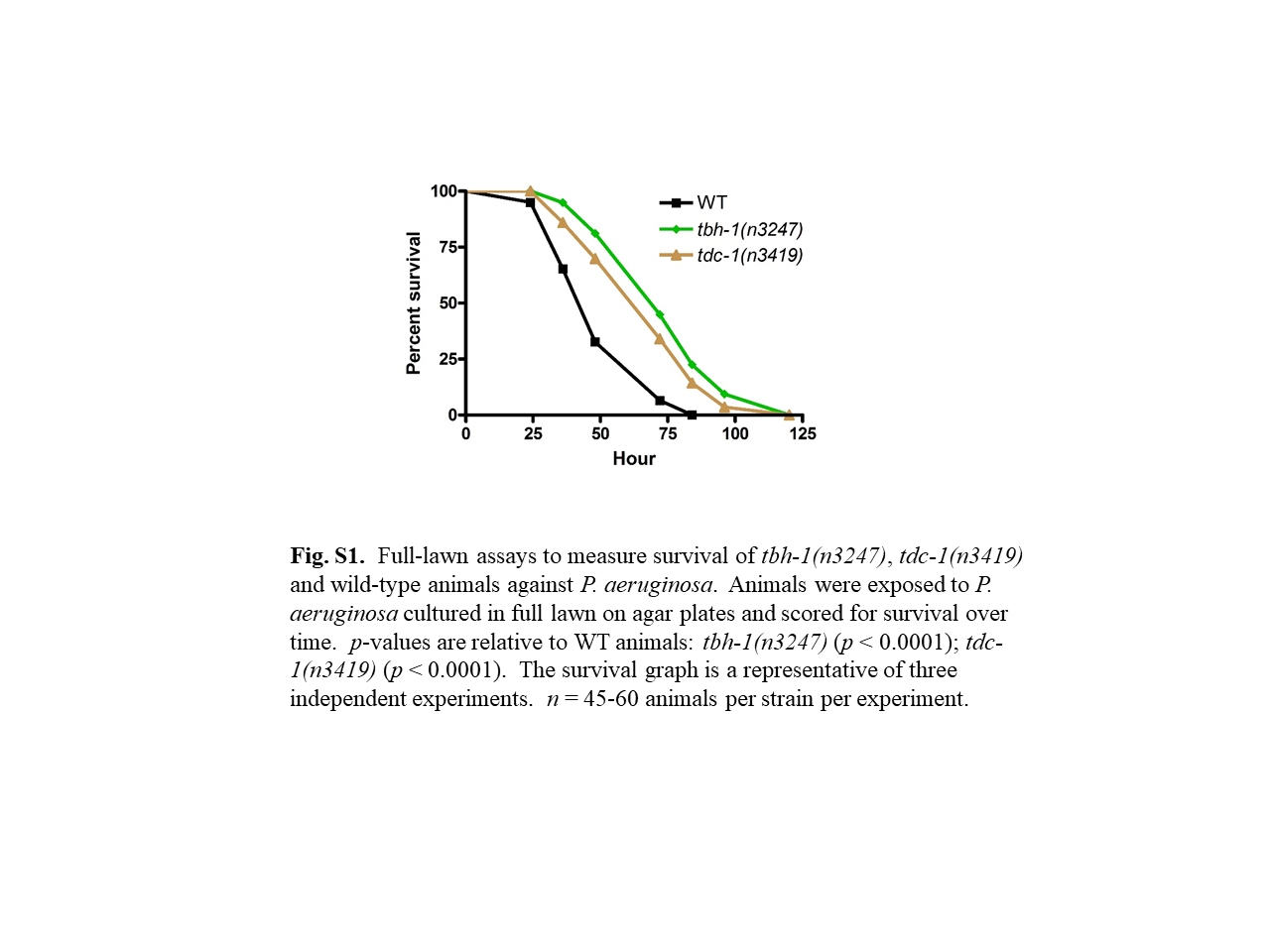

Supplement: FIG S1 [file mbo005184107sf1.tif]

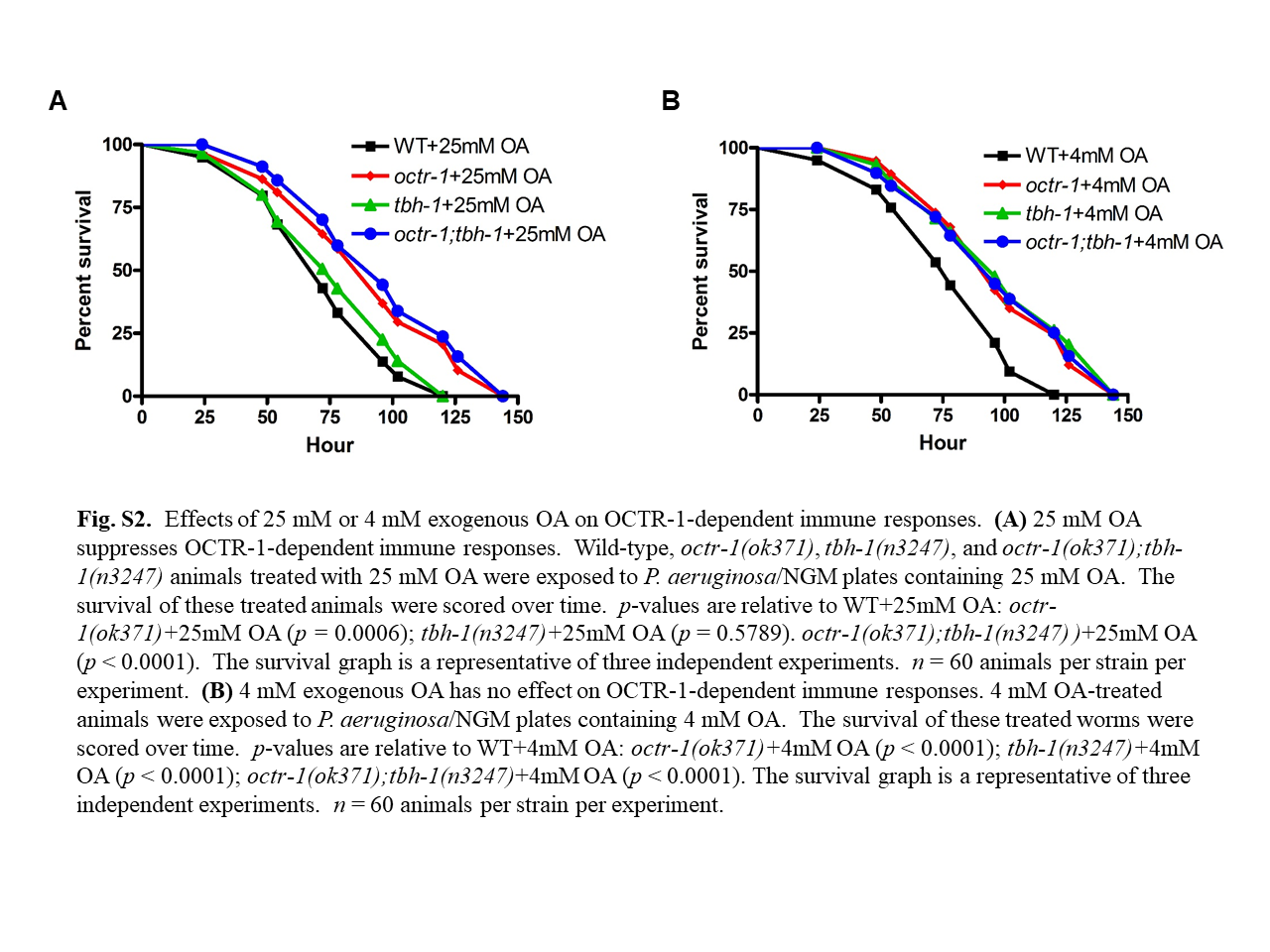

Supplement: FIG S2 [file mbo005184107sf2.tif]

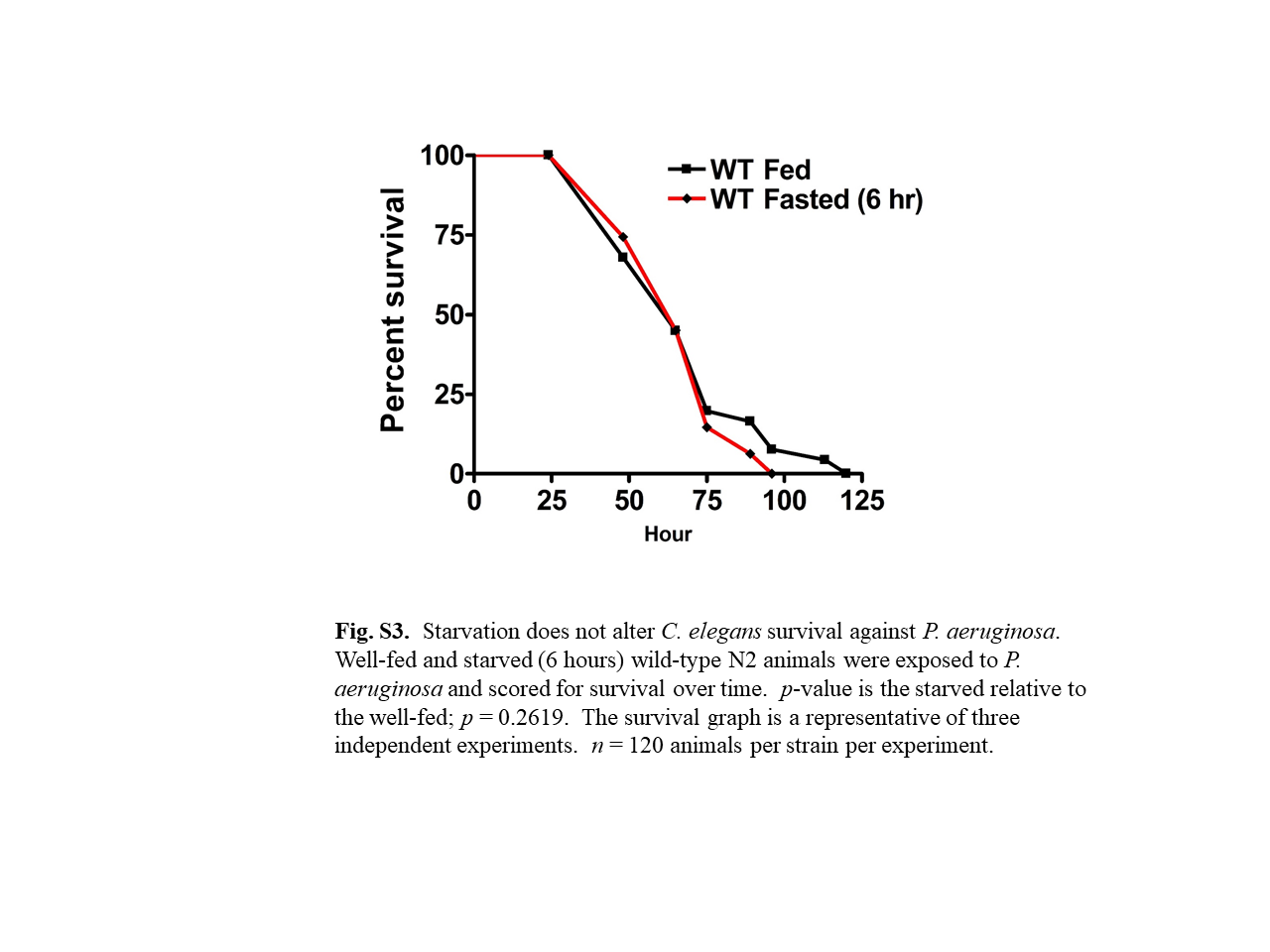

Supplement: FIG S3 [file mbo005184107sf3.tif]

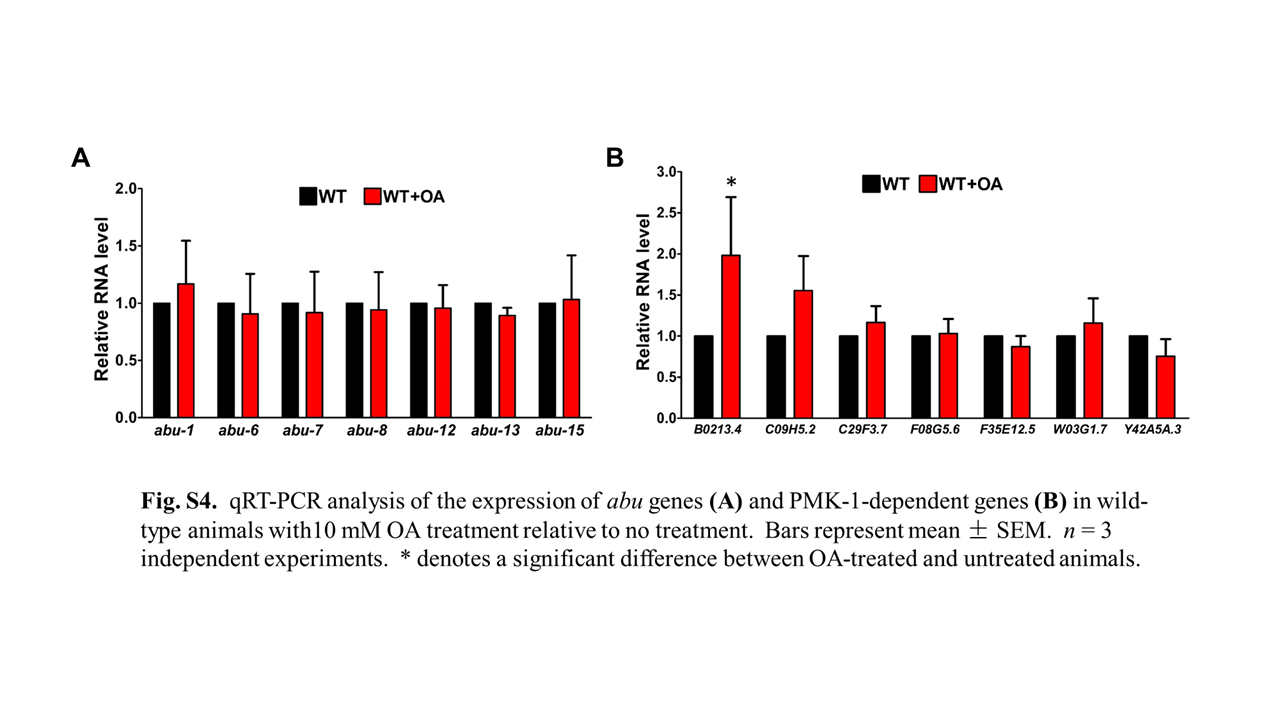

Supplement: FIG S4 [file mbo005184107sf4.tif]

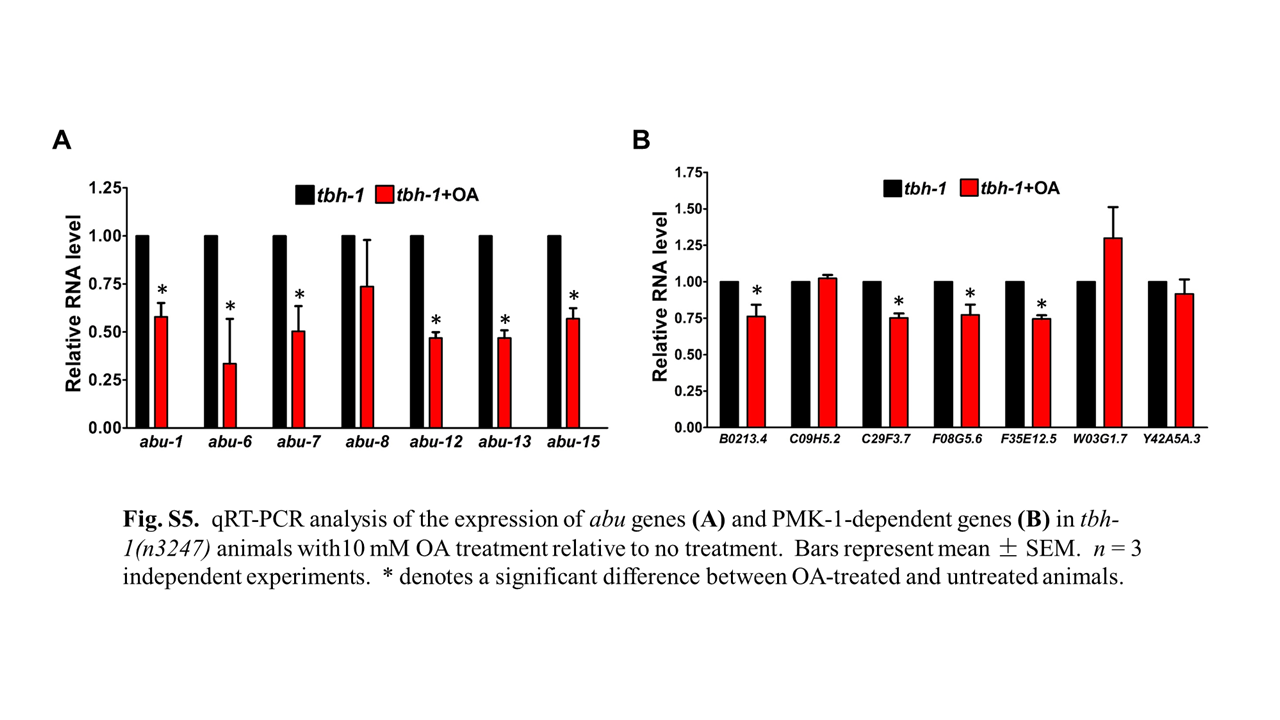

Supplement: FIG S5 [file mbo005184107sf5.tif]

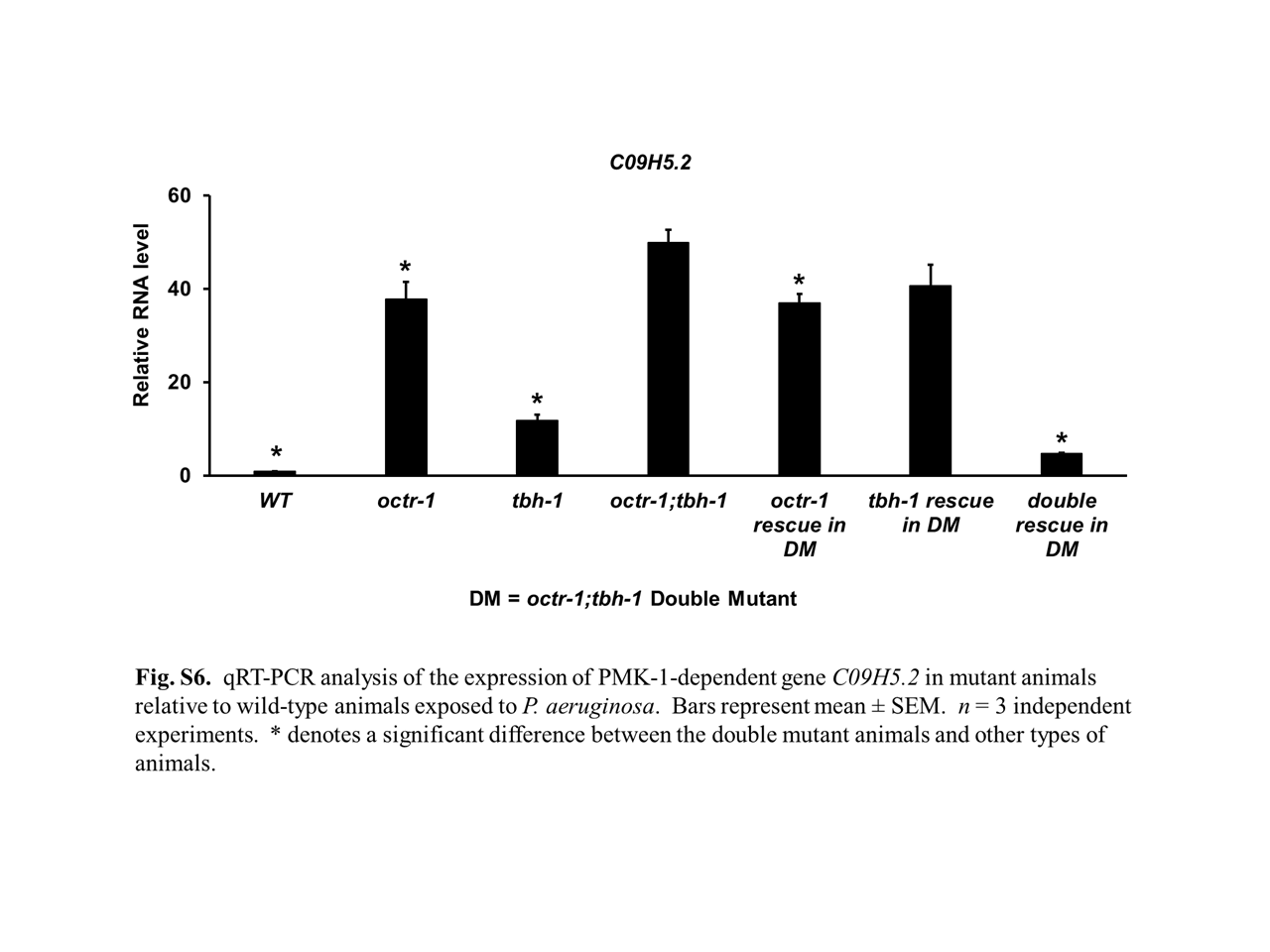

Supplement: FIG S6 [file mbo005184107sf6.tif]

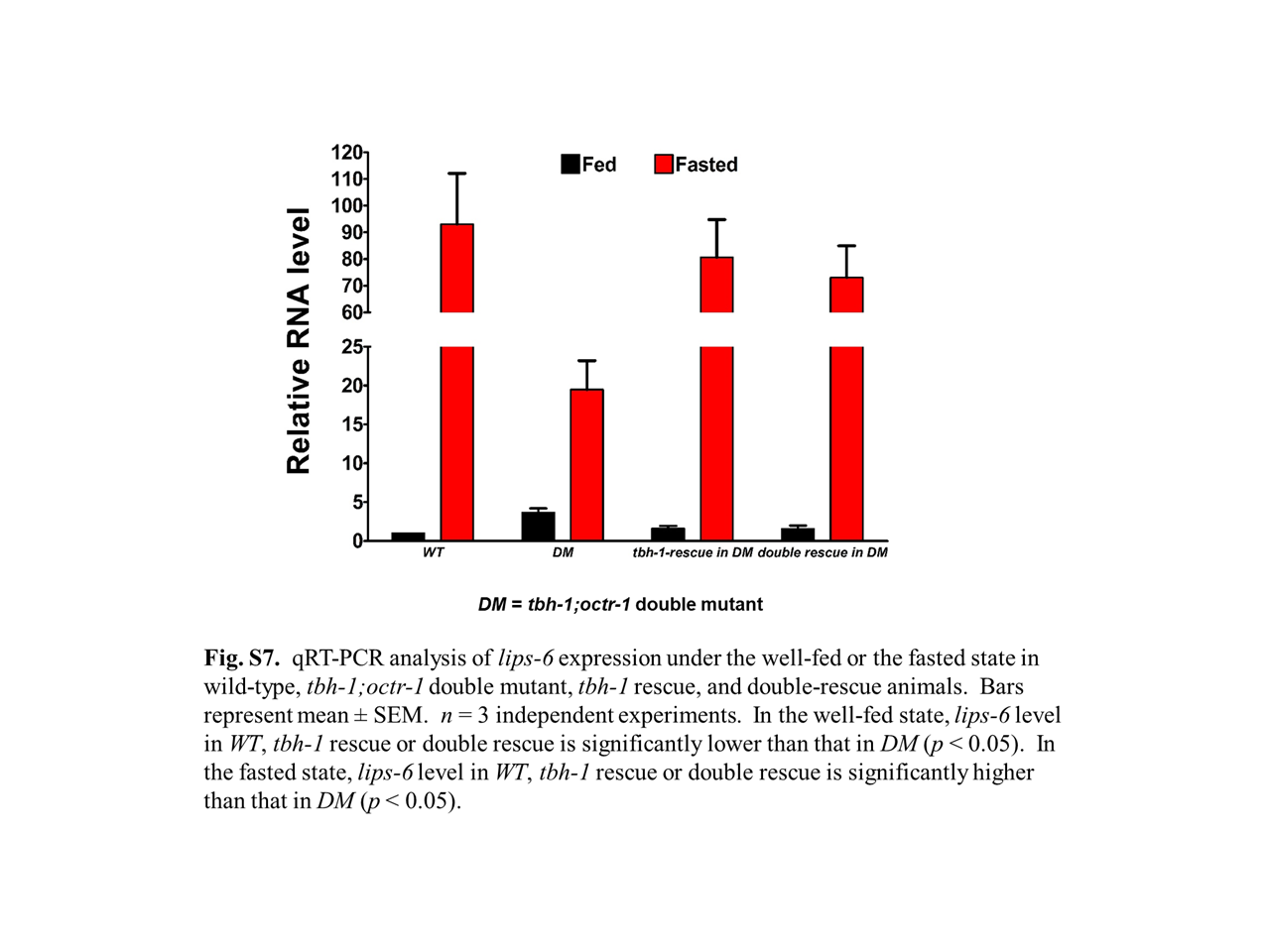

Supplement: FIG S7 [file mbo005184107sf7.tif]

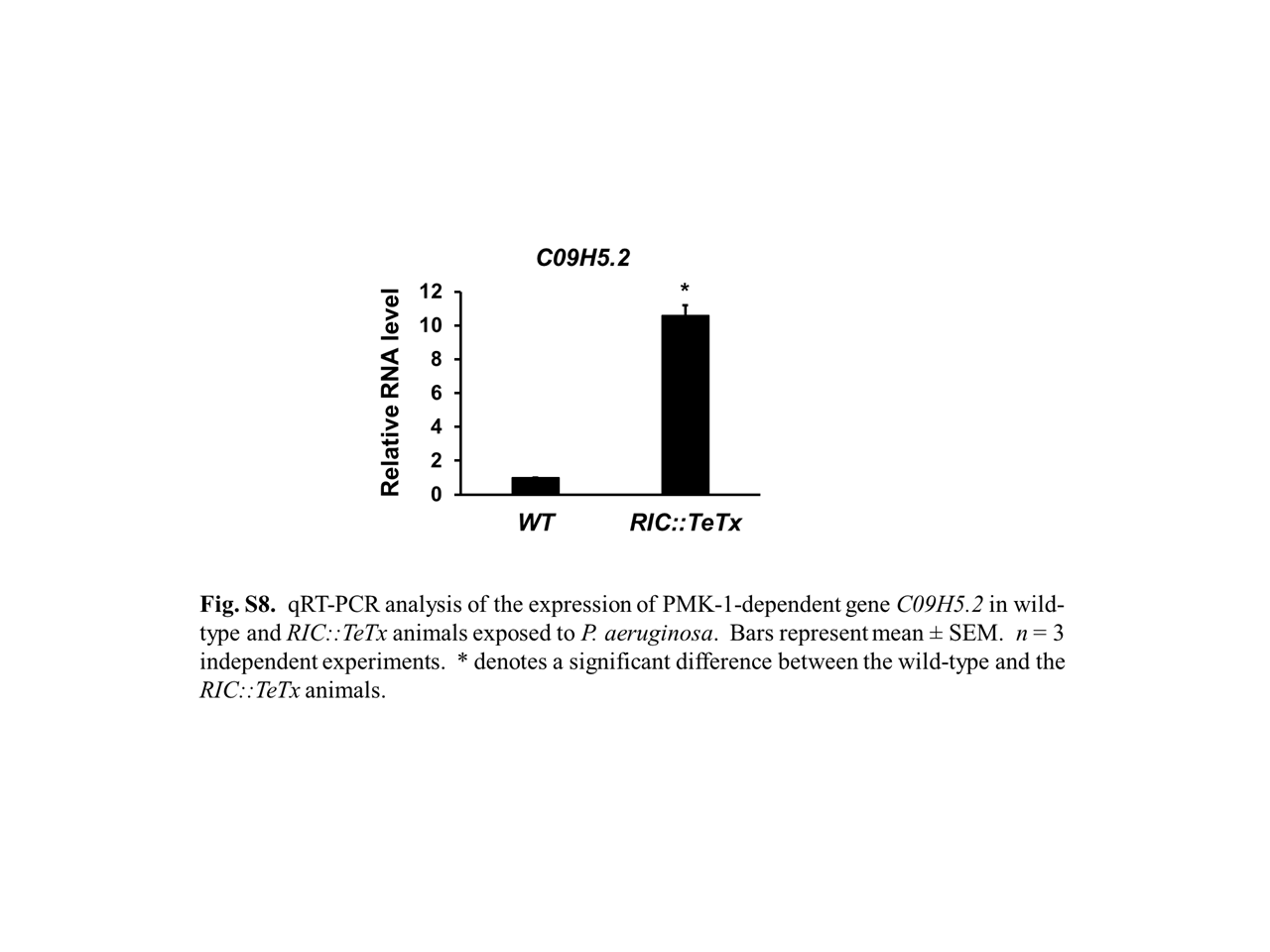

Supplement: FIG S8 [file mbo005184107sf8.tif]
