# Supplementary material for: Octopaminergic Signaling Mediates Neural Regulation of Innate Immunity in Caenorhabditis elegans
Source: mBio. 2018 Oct 9;9(5):e01645-18. doi: 10.1128/mBio.01645-18 (PMC6178624; doi:10.1128/mBio.01645-18)
Supplement: TABLE S1 [file mbo005184107s1.pdf]

Supplemental Methods

***C. elegans* Survival Assay.** *C. elegans* wild-type animals and mutants were maintained as hermaphrodites at 20°C and fed *E. coli* OP50 on modified nematode growth medium (NGM) agar plates (0.35% instead of 0.25% peptone) as described (S. Brenner, Genetics 77:71-94, 1974). The bacterial lawn used for *C. elegans* killing assays were prepared by placing a 25 μl drop of an overnight bacterial culture on modified NGM agar plates (3.5 cm diameter Petri plates). Plates were incubated at 37°C for 16 hours. Plates were cooled down at room temperature for at least 1 hour before seeding with synchronized animals. The survival assays were performed at 25°C and live animals were transferred daily to fresh plates. Animals were scored at the times indicated and were considered dead when they failed to respond to touch.

Pharyngeal Pumping Rate Assay. Wild-type, *tbh-1(n3247)*, and *tdc-1(n3419)* animals were synchronized by placing twenty gravid adult worms on NGM plates seeded with *E. coli* OP50 and allowing them to lay eggs for 45 minutes at 25°C. After which the gravid adult worms were removed and the eggs were allowed to hatch and grow at 20°C until they reached young adult stage. The synchronized worms were transferred to NGM plates fully seeded with *P. aeruginosa* for 24 hours at 25°C. Worms were observed under the Leica M80 microscope with focus on the pharynx. The number of contractions of the pharyngeal bulb was counted over 30 seconds. Counting was conducted in triplicate and averaged to give a pumping rate.

Defecation Rate Assay. Wild-type, *tbh-1(n3247)*, and *tdc-1(n3419)* animals were synchronized by placing twenty gravid adult worms on NGM plates seeded with *E. coli* OP50 and allowing

them to lay eggs for 45 minutes at 25°C. After which the gravid adult worms were removed and the eggs were allowed to hatch and grow at 20°C until they reached young adult stage. The synchronized worms were transferred to NGM plates fully seeded with *P. aeruginosa* for 24 hours at 25°C. Worms were observed under the Leica M80 microscope with a 60x magnification at room temperature. For each worm an average of 10 intervals between two defecation cycles was measured. The defecation cycle was identified as a peristaltic contraction beginning at the posterior body of the animal and propagating anteriorly followed by feces expulsion.

Bacterial Lawn Avoidance Assay. The behavioral avoidance assay was performed as described (J. Sun, V. Singh, R. Kajino-Sakanoto, and A. Aballay, Science 332:729-732, 2011). Briefly, small lawns of *P. aeruginosa* PA14 were cultured on 3.5cm NGM plates overnight at 37°C. Twenty young adult nematodes grown on *E. coli* OP50 were put in the center of each bacterial lawn. The number of nematodes was counted at 4, 8, 12, 24, 30 and 36 hours post-exposure for presence or absence on each lawn.

Profile of Bacterial Accumulation in the Nematode Intestine. To determine the profiles of bacterial accumulation in the intestine, wild-type, *tbh-1(n3247),* and *tdc-1(n3419)* animals were synchronized by placing twenty gravid adult worms on NGM plates seeded with *E. coli* OP50 and allowing them to lay eggs for 45 minutes at 25°C. After which the gravid adult worms were removed and the eggs were allowed to hatch and grow at 20°C until they reached young adult stage. The synchronized worms were transferred to NGM plates seeded with *P. aeruginosa/*GFP for 24 hours at 25°C. The animals were transferred to M9 buffer containing 25 mM sodium

azide and washed twice to eliminate *P. aeruginosa/*GFP stuck to the body of the nematodes. Animals were visualized using a Leica MZ10F fluorescence stereomicroscope.

Quantification of Intestinal Bacterial Loads. For the quantification of colony forming units (cfu), wild-type, *tbh-1(n3247),* and *tdc-1(n3419)* animals were synchronized by placing twenty gravid adult worms on NGM plates seeded with *E. coli* OP50 and allowing them to lay eggs for 45 minutes at 25°C. After which the gravid adult worms were removed and the eggs were allowed to hatch and grow at 20°C until they reached young adult stage. The synchronized worms were transferred to NGM plates seeded with *P. aeruginosa/*GFP for 24 hours at 25°C. The animals were transferred to M9 buffer containing 25 mM sodium azide and washed twice. The animals were then left to soak in a solution of 1mg/ml rifamycin, gentamycin and tetracycline for 1 hour. Ten nematodes per condition were transferred into 50 microliters PBS plus 0.1% Triton and ground. Serial dilutions of the lysates (10^{-1}, 10^{-2}, 10^{-3}, 10^{-4}) were plated onto LB/Ampicillin plates to select for *P. aeruginosa/GFP* cells and grown for 24 hours at 37°C.

RNA interference. RNA interference was conducted by feeding *C. elegans E. coli* strain HT115(DE3) expressing double-stranded RNA (dsRNA) that is homologous to a target gene (A.G. Fraser, R.S. Kamath, P. Zipperlen, M. Martinez-Campos, M. Sohrmann, and J. Ahringer, Nature 408:325-330, 2000; L. Timmons, and A. Fire, Nature 395:854, 1998). Briefly, *E. coli* with the appropriate vectors was grown in LB broth containing ampicillin (100 µg/ml) at 37^{0}C overnight, and plated onto NGM plates containing 100 µg/ml ampicillin and 3 mM isopropyl β-D-thiogalactoside (IPTG). RNAi-expressing bacteria were allowed to grow overnight at 37^{0}C. L2 or L3 larval animals were placed on RNAi or vector control plates for 2 days at 20^{0}C until

nematodes became gravid. Gravid adults were then transferred to fresh RNAi-expressing bacterial lawns and allowed to lay eggs for 1 hour to synchronize the second-generation RNAi population. The gravid adults were removed and eggs were allowed to develop at 20^{0}C to reach young adult stage for subsequent assays. Clone identity was confirmed by sequencing. *unc-22* RNAi was included as a positive control in all experiments to account for RNAi efficiency.

RNA isolation. Gravid adult animals were lysed using a solution consisting of sodium hydroxide and bleach at a volume ratio of 5:2. The eggs were washed, and synchronized for 22 hours in S basal liquid medium at room temperature. Synchronized L1 larval animals were placed onto NGM plates seeded with *E. coli* OP50 and grown at 20°C until the animals reached L4 stage. Animals were collected, washed with M9 buffer, transferred to NGM plates containing *P. aeruginosa* PA14, and then incubated for 4 hours at 25°C. After which animals were collected and washed with M9 buffer, and RNA was extracted using QIAzol lysis reagent (QIAGEN) and purified with RNeasy Plus Universal Kit (QIAGEN).

Quantitative real-time PCR. Total RNA was obtained as described above and subjected to reverse transcription using the High Capacity cDNA Reverse Transcription Kit (Applied Biosystems). Quantitative PCR with 50 ng cDNA per 25 μL reaction was conducted on StepOnePlus Real-Time PCR system using Power SYBR Green PCR Master Mix in a 96-well plate format (Applied Biosystems). Relative fold-changes for transcripts were calculated using the comparative C_T ($2^{-\Delta\Delta C_T}$) method and normalized to pan-actin (*act-1, -3, -4*). Cycle thresholds of amplification were determined by StepOne Software v2.3 (Applied Biosystems). All samples were run in triplicate. Primer sequences are available upon request.

Starvation of worms. To prepare starved animals for survival assays, young adult animals were placed onto NGM plates without bacteria food for six hours (S. Suo, Y. Kimura, and H.H. Van Tol, J Neurosci 26:10082-10090, 2006) before being transferred to *P. aeruginosa* lawn for survival assays. Specifically, wild-type worms were synchronized by placing twenty gravid adult worms on NGM plates seeded with *E. coli* OP50 and allowing them to lay eggs at 25°C for 45 minutes. Then the gravid adult worms were removed, and the eggs were allowed to hatch and grow at 20°C for 60 hours. Synchronized worms were collected and washed thrice with M9 buffer to remove any adhered bacteria. These worms were split into two groups: one group were transferred to NGM plates seeded with *E. coli* OP50 and the other group were transferred to unseeded NGM plate and incubated at 20°C for 6 hours. The former group are considered well fed and the latter starved. After incubation, worms were used for survival assays as described above.
