# Supplementary material for: Octopaminergic Signaling Mediates Neural Regulation of Innate Immunity in Caenorhabditis elegans
Source: mBio. 2018 Oct 9;9(5):e01645-18. doi: 10.1128/mBio.01645-18 (PMC6178624; doi:10.1128/mBio.01645-18)
Supplement: TEXT S1 [file mbo005184107st1.pdf]

Table S1. List of transgenic strains generated in this study

Strain	Plasmid	Host	Note
JRS1	*tbh-1p(4.5kb)::tbh-1 gDNA::mCherry*	*tbh-1(n3247)*	rescue TBH-1 in *tbh-1* mutant worm
JRS2	*tbh-1p(4.5kb)::tbh-1 gDNA::mCherry*	*octr-1(ok371);tbh-1(n3247)*	rescue TBH-1 in *octr-1;tbh-1* double mutant worm
JRS3	*octr-1p(4kb)::octr-1 cDNA::gfp*	*octr-1(ok371)*	rescue OCTR-1 in *octr-1* mutant worm
JRS4	*octr-1p(4kb)::octr-1 cDNA::gfp*	*octr-1(ok371);tbh-1(n3247)*	rescue OCTR-1 in *octr-1;tbh-1* double mutant worm
JRS5	*tbh-1p(4.5kb)::tbh-1 gDNA::mCherry and octr-1p(4kb)::octr-1 cDNA::gfp*	*octr-1(ok371);tbh-1(n3247)*	rescue both OCTR-1 and TBH-1 in *octr-1;tbh-1* double mutant worm
JRS6	*tbh-1p(4.5kb)::GCaMP6s*	N2	RIC::GCaMP6s
JRS8	*tbh-1p(4.5kb)::TeTx::mCherry*	N2	RIC-deficient worms
JRS9	*cat-1p::TU#813+tbh-1p::TU#814+coel::dsRed*	*MT9971[nIs107[tbh-1p::GFP+lin-15(+)]III]*	RIC-ablated worms
